# Supplementary material for: Reconstructing tissue culture to improve Agrobacterium‐mediated transformation of maize
Source: Plant J. 2026 Oct 23;128(2):e71183. doi: 10.1111/tpj.71183 (PMC13691924; doi:10.1111/tpj.71183)
Supplement: Supplementary file 1 [file TPJ-128-0-s001.pdf]

AH1 (hygromycin) **AG1 (G418)**

T0 plantlets

AG1 (G418)

T1 seedlings

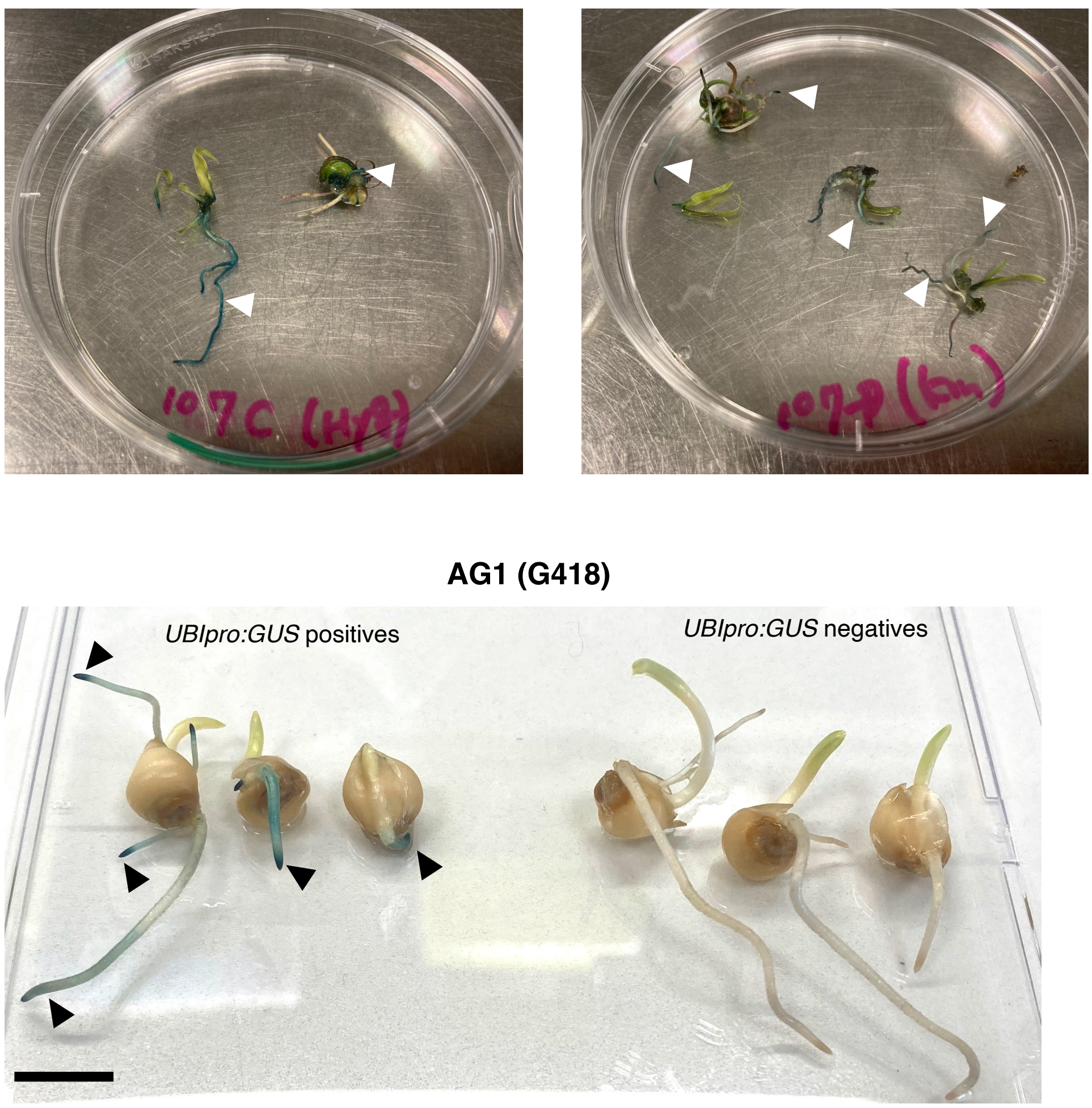

Supporting Figure S10
