# Supplementary material for: Reconstructing tissue culture to improve Agrobacterium‐mediated transformation of maize
Source: Plant J. 2026 Oct 23;128(2):e71183. doi: 10.1111/tpj.71183 (PMC13691924; doi:10.1111/tpj.71183)
Supplement: Supplementary file 9 [file TPJ-128-0-s010.pdf]

Supporting Experimental Procedure S1 (Methods S1)

A step-by-step protocol for *Agrobacterium*-mediated maize transformation

Table of contents:

1. **Preparation of stock solutions** **pp. 3-5**
2. **Preparation of stock media** **pp. 6-8**
3. **Preparation of solid working media** **pp. 9-11**
4. **Preparation of *Agrobacterium*** **pp. 12-13**
5. **Preparation of host maize plants and immature embryos** **pp. 14**
6. **Tissue culture procedures for transformation** **pp. 15-30**

    6.1. Embryo isolation, inoculation and co-culture (Day 1) **pp. 15-19**
    6.2. Embryo transfer to Resting medium: phase Resting (Day 4) **pp. 20**
    6.3. Embryo/Callus transfer to Sel-1 medium: phase Sel-1 (Day 14) **pp. 21**
    6.4. Callus transfer to Sel-2 medium: phase Sel-2 (Day 28) **pp. 22**
    6.5. Callus transfer to Sel-3 medium: phase Sel-3 (Day 42) pp. 23
    6.6. Callus transfer to Reg1 medium: phase Reg1-1 (Day 56) **pp. 24**
    6.7. Callus transfer to Reg1 medium: phase Reg1-2 (Day 66) **pp. 25**
    6.8. Callus transfer to Reg2 medium: phase Reg2p (Day 76) **pp. 26-27**
    6.9. Shoot/Callus transfer to Reg2 bottles: phase Reg2b (Day 83) **pp. 28-29**
    6.10. Plantlet transfer to soil trays or pots: phase Acclimatization (Day 93) **pp. 30**

7. **Figures and figure legends for Supporting Experimental Procedure 1 (SP1-Figs)** **pp. 31-44**

1. Preparation of stock solutions:

- Acetosyringone (25 mg/mL)

Acetosyringone	0.25 g	(Merck: Cat. No., D134406-5G)
Dimethyl Sulfoxide	to 10 mL	(Roth: Cat. No., 4720.1)

0.22 µm filter sterilization (syringe cap), 500 µL aliquot in sterile 1.5 mL microtubes, Store at -20 ºC.

- $AgNO_3$ (50 mM)
(making in and store in 50 mL falcon tube)

Silver nitrate	0.339 g	(PanReacAppliChem: Cat. No., A3944,050)
ddH_2O	to 40 mL	

0.22 µm filter sterilization (syringe cap), Store at 4 ºC, in the dark condition (wrap the tube with aluminum-foil)

- L-Cystein (15 mg/mL)

L-Cystein	0.3 g	(Merck: Cat. No., C8755)
ddH_2O	to 20 mL	

0.22 µm filter sterilization (syringe cap), make 1 mL aliquots in sterile 1.5 mL test tubes, Store at -20 ºC. One-time use and avoid freeze-thaw cycles.

- Dicamba (30 mM)
(making in and store in 50 mL falcon tube)

Dicamba	0.25 g	(Duchefa: Cat. No., D0920.250)
Absolute Ethanol	19 mL	(VWR: Cat. No., 20821.330)
(Dissolve powder completely)		
ddH_2O	19 mL	

0.22 µm filter sterilization (syringe cap), Store at 4 ºC.

- Myo-inositol (50 mg/mL)
(making in and store in 50 mL falcon tube)

Myo-inositol 2.5 g (Duchefa: Cat. No., I0609)
ddH$_2$O to 50 mL

0.22 µm filter sterilization (syringe cap), Store at 4 ºC.

- Meropenem trihydrate (12.5 mg/mL)

Meropenem trihydrate 1 g (FUJIFILM Wako: Cat.No., 133-15671)
ddH$_2$O (warmed up to 40 ºC) to 80 mL

0.22 µm filter sterilization (syringe cap), make 1 mL aliquots in sterile 1.5 mL test tubes, Store at -20 ºC. Avoid extensive freeze-thaw cycles.

- 2,4-D (1 mg/mL)

(making in and store in 50 mL falcon tube)

2,4-D 50 mg (Merck: Cat. No., D8407)
Absolute Ethanol 2.5 mL
(Dissolve powder completely)
ddH$_2$O to 50 mL

0.22 µm filter sterilization (syringe cap), Store at 4 ºC.

- N6/MS Vitamins (x 1000)

(store in sterilized bottles)

Glycine	2 g	(Merck: Cat. No., G7126)
Thiamin HCl	0.5 g	(Duchefa: Cat. No., T0614)
Pyridoxin	0.5 g	(Duchefa: Cat. No., P0612)
Nicotinic acid	0.05 g	(Duchefa: Cat. No., N0611)
ddH$_2$O	to 1 L	

0.2 µm filter sterilization (bottle top), make aliquots in smaller sterile bottles, store at 4 ºC

- 1/10 BASTA solution (1.35 % Glufosinate)

BASTA stock solution (13.5 % Glufisinate) 1 mL (BASF)
ddH$_2$O 9 mL

0.22 µm filter sterilization (syringe cap), make 500 µL aliquots in sterile 1.5 mL test tubes, Store at -20 ºC

- Glufosinate ammonium stock (10 mg/mL)

Glufosinate ammonium	100 mg	(Merck: Cat. No., 45520-100MG)
ddH_2O	to 10 mL	

0.22 µm filter sterilization (syringe cap), make 500 µL aliquots in sterile 1.5 mL test tubes, Store at -20 ºC. Avoid extensive freeze-thaw cycles.

- Hygromycin B stock (50 mg/mL)

Hygromycin B conc. solution (500 mg/ml)	1 mL	(Duchefa: Cat. No., H0192.0001)
ddH_2O	to 10 mL	

0.22 µm filter sterilization (syringe cap), make 500 µL aliquots in sterile 1.5 mL test tubes, store at -20 ºC. Avoid extensive freeze-thaw cycles.

- G418 stock (50 mg/mL)

Make 500 µL aliquots in sterile 1.5 mL test tubes, from
Geneticin disulphate (G418) solution (50 mg/mL) (Carl Roth: Cat. No., 2039.1)
Store at -20 ºC. Avoid extensive freeze-thaw cycles.

- 5 % silwet solution

Silwet® L-77	250 µL	(plantMedia: Cat. No., 30630216-3)
ddH_2O	4.75 mL	

0.22 µm filter sterilization (syringe cap), make 500 µL aliquots in sterile 1.5 mL test tubes, store at room temperature in the dark condition.

- 3 % bleach solution

Sodium hypochlorite 13 %	230 mL	(PanReacAppliChem: Cat. No., 213322.1214)
deionized H_2O	to 1 L	
Lipsol® Detergent	2 mL	(SciLabWare)

Mix well and store at -4 ºC

2. Preparation of stock media:

- 1x Infection Medium liquid stock

N6 Basal Salts	3.95 g	(Duchefa: Cat. No., C0203.0050)
N6/MS Vitamins (x 1000)	1 mL	
Proline	0.7 g	(Duchefa: Cat. No., P0717.0100)
2,4-D (1 mg/mL)	1.5 mL	
Sucrose	68.4 g	(Duchefa: Cat. No., S0809.5000)
Glucose (monohydrate)	39.6 g	(Duchefa: Cat. No., G0802.5000)
deionized H_2O	to 1 L	

pH=5.2 with HCl/NaOH, 0.2 µm filter sterilization (bottle top), make aliquots in smaller sterile bottles, store at 4 ºC

- 2x CoCult liquid stock

MS Basal Salts	8.6 g	(Duchefa: Cat. No., M0221.0100)
N6/MS Vitamins (x 1000)	2 mL	
Sucrose	60.0 g	
Myo-inositol (50 mg/mL)	4 mL	
Casamino acids	0.2 g	(Merck: Cat.No., C4523)
Proline	1.4 g	
Dicamba (30 mM)	1 mL	
deionized H_2O	to 1 L	

pH=5.8 with KOH, 0.2 µm filter sterilization (bottle top), make aliquots in 500 mL sterile bottles, store at 4 ºC

- 2x Rest/Sel liquid stock

MS Basal Salts	25.8 g	
N6/MS Vitamins (x 1000)	6 mL	
Dicamba (30 mM)	3 mL	
Sucrose	180.0 g	
MES	3 g	(PanReacAppliChem: Cat. No., A0689,050)

Myo-inositol (powder)	0.6 g
Casamino acids	0.6 g
Proline	4.2 g
deionized H_2O	to 3 L

pH=5.8 with KOH, 0.2 µm filter sterilization (bottle top), make aliquots in 500 mL sterile bottles, store at 4 ºC

- 2x Reg1 liquid stock

MS Basal Salts	25.8 g
N6/MS Vitamins (x 1000)	6 mL
Sucrose	360.0 g
Myo-inositol (powder)	0.6 g
deionized H_2O	to 3 L

pH=5.8 with KOH, 0.2 µm filter sterilization (bottle top), make aliquots in 500 mL sterile bottles, store at 4 ºC

- 2x MS^- liquid stock

MS Salts (with Vitamin)	26.4 g	(Duchefa: Cat. No., M0222.0050)
Sucrose	180.0 g	
deionized H_2O	to 3 L	

pH=5.8 with KOH, 0.2 µm filter sterilization (bottle top), make aliquots in 500 mL sterile bottles, stored at 4 ºC

- 2x 0.23 % Phytagel stock

In a 500 mL medium bottle:

Phytagel	1.15 g	(Merck: Cat. No. P8169-500G)
deionized H_2O	250 mL	

Carefully dissolve phytagel powder to prevent massive aggregation, mix well and equilibrate for an overnight, autoclave and store at room temperature

- 2x 0.3 % Phytagel stock

In a 500 mL medium bottle,

Phytagel	1.5 g
deionized H_2O	250 mL

Carefully dissolve phytagel powder to prevent massive aggregation, mix well and equilibrate for an overnight, autoclave and store at room temperature

3. Preparation of solid working media:

➢ ***General procedures***:

1) Warm liquid stock medium up to room temperature before use.
2) Boil phytagel stocks in a storage bottle using microwave or a heating device.
3) Mix boiled phytagel stock and liquid stock, cool the mixture down approximately at 60 °C, then add supplements and mix well.
4) Pour mixtures into dishes/bottles and solidify medium with lids open to prevent excessive water drop formation.

➢ CoCult plates for co-culture

(Mix followings in a 100 mL flat-bottom flask)

2x CoCult liquid stock	25 mL
2x 0.23 % Phytagel stock	25 mL
$AgNO_3$ (50 mM)	90 µL
L-Cysteine (15 mg/mL)	1 mL
Acetosyringone (25 mg/mL)	50 µL

Pour in 50 mm ø dishes (6 plates for 50 mL), solidify for 10 min. Seal with parafilm and store at 22 °C. These plates can be stored for maximum 5 days.

➢ Resting/Selection plates

(Mix followings in a 500 mL bottle in which phytagel stock stored)

● For Basta/glufosinate selection system

Type of medium plates	Resting	Sel-1	Sel-2	Sel-3
2x Rest/Sel liquid stock	250 mL	250 mL	250 mL	250 mL
2x 0.23 % Phytagel stock	250 mL	250 mL	250 mL	250 mL
$AgNO_3$ (50 mM)	900 µL	900 µL	900 µL	900 µL
Meropenen trihydrate (12.5 mg/mL)	1 mL	1 mL	1 mL	1 mL
1/10 BASTA solution	-	37.5 µL	75 µL	-
Glufosinate ammonium stock (10 mg/mL)	-	-	-	500 µL

Pour in 90 mm ø dishes (20 plates for 500 mL), solidify for 30 min. Seal with parafilm and store at 4 °C. These plates can be stored for maximum 4 months.

- For Hygromycin selection system

Replace 1/10 BASTA solution or Glufosinate ammonium stock to Hygromycin B stock as follows.

Type of medium plates	Resting	Sel-1(H)	Sel-2(H)	Sel-3(H)
Hygromycin B stock (50 mg/mL)	-	150 µL	300 µL	400 µL

- For G418 selection system

Replace 1/10 BASTA solution or Glufosinate ammonium stock to G418 stock as follows.

Type of medium plates	Resting	Sel-1(G)	Sel-2(G)	Sel-3(G)
G418 stock (50 mg/mL)	-	100 µL	200 µL	400 µL

- Reg-1 plates
- For Basta/glufosinate selection system

(Mix followings in a 500 mL bottle where phytagel stock stored)

2x Reg1 liquid stock	250 mL
2x 0.3 % Phytagel stock	250 mL
Meropenen trihydrate (12.5 mg/mL)	1 mL
1/10 BASTA solution	100 µL

Pour in 90 mm ø dishes (20 plates for 500 mL), solidify for 30 min. Seal with parafilm and store at 4 ºC. These plates can be stored for maximum 4 months.

- For Hygromycin selection system

Replace 1/10 BASTA solution to Hygromycin B stock as follows.

Type of medium plates	Reg1(H)
Hygromycin B stock (50 mg/mL)	400 µL

- For G418 selection system

Replace 1/10 BASTA solution to G418 stock as follows.

Type of medium plates	Reg1(G)
G418 stock (50 mg/mL)	400 µL

- Reg-2 plates and bottles
- For Basta/glufosinate selection system

(Mix followings in a 500 mL bottle where phytagel stock stored)

2x MS liquid stock	250 mL
2x 0.3 % Phytagel stock	250 mL
Myo-inositol (50 mg/mL)	1 mL
Meropenen trihydrate (12.5 mg/mL)	1 mL
1/10 BASTA solution	100 µL

(Reg2p) Pour in 90 mm ø x 20 mm height dishes (20 plates for 500 mL), solidify for 30 min. Seal with parafilm and store at 4 ºC. These plates can be stored for maximum 4 months.

(Reg2b) Pour in Beck 500 mL glass jars for 20 mm medium thickness (8 jars for 500 mL), solidify for 30 min. Store at 4 ºC. These bottles can be stored for maximum 4 months.

- For Hygromycin selection system

Replace 1/10 BASTA solution to Hygromycin B stock as follows.

Type of medium plates	Reg2(H)
Hygromycin B stock (50 mg/mL)	400 µL

- For G418 selection system

Replace 1/10 BASTA solution to G418 stock as follows.

Type of medium plates	Reg2(G)
G418 stock (50 mg/mL)	250 µL

4. Preparation of *Agrobacterium*:

- Host strain: LBA4404 harboring *pTiBo542*, which is maintained with YEP-hS supplemented with 10 mg/L of tetracycline.

- Medium: YEP-hS medium

Peptone	10 g	(Duchefa: Cat. No., P1328.1000)
Yeast Extract	5 g	(Duchefa: Cat. No., P1333.1000)
NaCl	1g	(Duchefa: Cat. No., S0520.5000)
deionized H_2O	to 1L	

pH=6.8 with KOH, make 250 mL aliquots in 500 mL bottles.
(For solid medium) Add 4 g of agar to each 250 mL medium (1.6 %).
Autoclave and store at room temperature.

- LBA4404(*pTiBo542*) competent cell preparation

Streak and pick up single colony of LBA4404(*pTiBo542*) from the glycerol stock appeared on YEP-hS (10 mg/L tetracycline, Merck: Cat. No., 87128) plate, make seed culture in 3 mL of YEP-hS liquid (10 mg/L tetracycline) for an overnight, at 28 ºC, 200 rpm.

Scale up seed culture to 200 mL of YEP-hS liquid (10 mg/L tetracycline) in a 1 L flat bottom flask, culture for approximately 6 hours at 28 ºC, 200 rpm, until OD_{600} reaches to 0.6-0.8.

Collect cells in 4x 50 mL falcon tubes, centrifuge at 4 ºC, 3000 g for 10 min.

Discard supernatant, resuspend cell pellets in 12.5 mL each of ice-chilled ddH_2O, collect resuspended cells into one 50 mL tube. Centrifuge at 4 ºC, 3000 g for 10 min.

Discard supernatant, resuspend cell pellets in 50 mL of ice-chilled ddH_2O. Centrifuge at 4 ºC, 3000 g for 10 min.

Discard supernatant, resuspend cell pellets in 10 mL ice-chilled 10 % glycerol solution (sterilized).

Make 100 µL aliquots in 1.5 mL sterile test tubes. Snap freeze aliquots with liquid nitrogen and store

at -80 ºC. Cells can keep high competency for 1 to 2 years.

- Transformation of *Agrobacterium* via electroporation.

Add 200-500 ng of your binary vector into 100 µL of competent cells and dissolve mixture well by carefully pipetting up and down on ice.

Pour all mixtures to pre-chilled electroporation cuvettes (2 mm gap), mind to not form bubbles in the gap.

Perform electroporation (1700 V, 6.0 ms) and place the cuvette back on ice immediately.

Resuspend and recover cells by adding 300 µL of YEP-hS liquid (without antibiotics, under the sterile hood) to the cuvette and transfer the suspension back to the 1.5 mL test tubes where competent cells were.

Incubate recovered cells at room temperature (~28 ºC), for 1 hours in the dark condition (no shaking required).

Spread recovered cells on the YEP-hS plate (8 mg/L tetracycline and 80 mg/L spectinomycin, Duchefa: Cat. No., S0188.0025, when the binary vector has $Spec^R$ gene for bacterial selection). Incubate cells at 26 ºC for 3 overnights.

Perform colony PCR to confirm positive colony and make the glycerol stock by culturing positive clone in 2 mL of YEP-hS liquid (8 mg/L tetracycline and 80 mg/L spectinomycin) for an overnight.

3 days prior to the maize transformation, streak approximately 50 µL of glycerol stock to the YEP-hS plate (8 mg/L tetracycline and 80 mg/L spectinomycin), incubate at 22-26 ºC for 2 overnight.

(optional) Use the transformed cell spread directly for the maize transformation by picking up bulk colonies as heterogenous bacterial cultures (usually, more than 90 % of *Agrobacterium* colonies are positive for the construct).

5. Preparation of host maize plants and immature embryos:

Prepare and culture wild type maize inbred line or lines of interest, according to the way described in Materials and Methods. Prepare at least 4-6 plants when you are willing to obtain 2-4 pollinated cobs. One cob will contain 150-250 and 200-300 kernels in A188 and B104, respectively, when pollination works perfectly.

Cover first cob before emergence of silks to prevent outcrosses at undesired timing. Do not use cobs at second position or below.

After 5-7 days of first silk emergence, cut silks at 3-5 cm from top of bract and perform pollination with enough amount of fresh pollen grains (collected from tassels having anthesis in 60-90 % of spikelet perform well). Cover the crossed cobs, labeling with genotype, pollination date and time.

After 10 to 13 days from pollination (depending on inbred line and growing condition), check the embryo size by opening a small window at the top of cob and picking up few developing kernels (SP1-Fig. 1A-D). If the embryos fit to the applicable size window, harvest cob and peal bracts with leaving inner most one or two layers. Store the harvested cobs in the fridge by sandwiching them between chilled cooling-gel pads (SP1-Fig. 1E). Mind that embryos will experience additional growth to some extent (~20% of vertical length in 3 days) in the fridge condition. The cobs cannot be stored longer than 5 days in the fridge (the competency of embryos will drop down after 5 days).
If the embryos are still small, seal the window with surgical tape and let them grow for few more days (SP1-Fig. 1F). The embryos will experience the growth from 0.4 to 0.6 mm in vertical length per 1 day in case of A188 inbred with well-controlled growth condition.

6. Tissue culture procedures for transformation

6.1. Embryo isolation, inoculation and co-culture (Day 1)

Before you start:

- Check if your *Agrobacterium* spread is growing well. In case of LBA4404(*pTiBo542*), bacteria culture showing yellowish-white colored spread/colonies performs better, while bacteria culture is getting old when it starts to show reddish colors.
- Check if you prepared enough number of CoCult plates and if there are no bacterial/fungus contamination.

- Tools (all sterilized accordingly, autoclaved or use fresh sterile products)
  (also refer SP1-Fig. 2 for tools and workspace set up under the sterile hood)

2x Scalpel blades with holders (type 11)
2x preparation knife (Hammacher: Cat. No., HWB202-20)
1x angled tweezers, 15 cm length (to pick up test-tubes and other items)
1x straight tweezers, 10 cm length (to pierce and fold maize cobs)
1x straight tweezers, 20 cm length (to handle cobs during surface sterilization and rinsing steps)

2 mL sterilized test tubes
Test tube holder
50 mL falcon tubes (sterilized)
Falcon tube holder

Pasteur glass pipettes (with cotton filter, sterilized)
Rubber for Pasteur pipettes

Micropipettes for 1000 µL, 100 µL and 20 µL
Sterilized pipet tips for 1000 µL and 20-100 µL

1 L (or bigger) sterilized plastic or glass beaker (to perform cob sterilization and rinse), with covering aluminum foil
3 L plastic beaker (for waste solutions for cob sterilization and rinse)
100 mL bottle (for bacterial waste solution)
Sterilized wider petri dishes, round or square, to perform dissection on it.

- Solutions and medium

3 % bleach solution (+ 0.2 % Libsol, stored at 4 ºC)
ddH2O (for rinsing cobs after sterilization, approximately 500 mL - 1 L for 1 cob)
70 % Ethanol in wash bottle (for surface sterilization of cob, tools and hands)
70 % Ethanol in a 100 mL medium bottle (for sterilization of scalpel blades and knives)
1x Infection Medium liquid stock (at room temperature before use)
Acetosyringone (25 mg/mL, thaw in the dark condition at room temperature before use)
CoCult plates (keeping in dark condition)
5 % Silwet stock solution (keeping in dark condition)

- Equipment and devices

Binocular microscopy (surface sterilize and placing under the sterile hood)
Flaming device such as gas burner (placing under the sterile hood, with a lighter if needed)
Shaker (benchtop)
Vortex mixer (benchtop)
Spectrophotometer and corresponding cuvettes (benchtop)
Incubation chamber set at 22 ºC and dark condition.

- Procedure and tips

  (also refer SP1.Fig.3 for the work-flow illustration of this procedure)

6.1.1. Surface-sterilize harvested cobs by wiping with 70 % ethanol, peal the remaining bracts and remove silks and unnecessary parts of the cob. Place cob in the sterile beaker. If cob is too big for the washing beaker, break the cob into two parts at the middle (however this will be difficult for B104 cob due to its hardness). Pour 300-400 mL of 3 % Bleach solution until cobs are fully covered. Put covering aluminum foil on the beaker and shake at 100-150 rpm for 20 min. making sure that the bleach solution is covering cob over and over while shaking.

6.1.2. During cob sterilization, prepare working Infection Medium (wIM), by measuring 40 mL 1x Infection Medium stock medium in a 50 mL falcon tube (use scales marked on falcon tube), adding 80 µL of Acetosyringone (25 mg/mL) and mixing vigorously. 40 mL of wIM is corresponding to the amount to perform transformation of 2 constructs using 70-130 embryos each yielded from one cob.

6.1.3. Aliquot wIM as follows and label accordingly (per construct):

4 mL in a 50 mL falcon tube (is for *Agrobacterium* suspension, scaled up)

1 mL in a 2 mL test tube (is for *Agrobacterium* suspension, first resuspension)

2 mL in a 2 mL test tube (is for immature embryo collection)

An empty 2 mL test tube (is for the collection Agrobacterium suspension after inoculation)

Also prepare wIM as follows per cob (embryo population):

5 mL in a 50 mL falcon tube (is for working silwet solution)

990 mL in a 2 mL test tube and add 10 µL of 5% silwet solution (is now 0.05 % silwet intermediate)

6.1.4. After 20 min. of cob sterilization, discard 3% bleach solution (collect and discard the waste accordingly). Pour 200 mL of ddH_2O and rinse cobs with gentle shaking. Discard water and repeat this rinsing step once more. Pour 300-400 mL of ddH_2O until cobs are fully covered. Put covering aluminum foil and shake them at 100-150 rpm for 5 min. making sure that the rinsing water is covering cob over and over while shaking.

6.1.5. After 5 min of rinsing, discard water and pick up cobs, and pierce cobs with a short straight tweezer (Be careful to not injure your fingers and hands).

Start embryo isolation. To do this, first cut developing kernels horizontally using scalpel blade at one-third position from the top (perform this for 2-4 rows at once). Then using knife, remove cellularizing endosperm by pulling kernels little bit to bottomward and scooping. Now you may find that an embryo remains inside the kernel. Sometimes embryo may be attaching to the scooped endosperm tissue, so that carefully check this possibility too. Scoop up embryo and transfer to the collection tube with 2mL wIM. Change scalpel blade and knife with sterilizing by flame and 70 % ethanol every 2-4 lows (you have two sets of blade and knife for rotating use). When you are working with 2 or more constructs to transform, distribute embryos into two or multiple collection tubes. Finish or stop this embryo collection work in 1 hour (maximum 75 min.). After finishing embryo collection, close test tube lid and invert tubes several times, to make embryos sink to the bottom. Also check embryo size and morphology during collection to determine strength of silwet treatment according to Fig. 3B.

6.1.6. After embryo collection, make *Agrobacterium* suspension in wIM. To do this, first collect *Agrobacterium* cells from culture on YEP-hS plate on the tip of a 1000 µL micropipette tip (approximately 2 mm diameter). Dissolve these cells in the 2 mL test tube with 1 mL of wIM. Particularly, LBA4404 cells are too sticky to make resuspension directly into the liquid,

therefore, first spread cells on the side of test tube and then dissolve gradually by adding small amount of wIM, and in the end, carefully pipet up and down to make the suspension completely homogenized. Next, you transfer 1 mL of resuspended Agrobacterium to the 4 mL wIM medium prepared in the 50 mL falcon tube and mix well with gentry shaking tubes horizontally.

6.1.7. Take 1 mL from scaled-up Agrobacterium suspension for the OD_{600} measurement with the spectrophotometer. Add corresponding amount of wIM to adjust it to $OD_{600} = 0.4$, according to the equation below. For example, if your Agro-suspension showed $OD_{600} = 0.65$, add 2.5 mL of wIM.

(Equation) $Y = 10 * X - 4$

X is measured OD_{600} value of your agro-suspension while Y is the amount of wIM (mL) to be added.

6.1.8. Prepare working Silwet solution according to the size and morphology of embryos you collected. For example, if you are willing to make 0.005 % Silwet solution, discard 500 µL of wIM from 5 mL, then add 500 µL of 0.05 % silwet intermediate.

6.1.9. Prepare capillary Pasteur pipettes for number of constructs plus one. Not necessary to make them extremely thin, however make them thinner than embryo size to prevent accidental sucking up of embryos during washing step. You may able to prepare these capillary pipettes before starting embryo isolation (in this case, store capillary pipettes carefully to not injure your fingers and hands while embryo collection).

6.1.10. Using capillary Pasteur pipette, carefully suck up and discard wIM from the embryo collection tube. You can use same capillary Pasteur pipette across multiple tubes, as there is no danger of cross contamination yet. Add 1 mL of working Silwet solution using micropipette and close the lid. Perform 1 second flash vortex for 10 times with 2 times inverting in the intervals.

6.1.11. Using capillary Pasteur pipette (you can use same one from step 6.1.11.), carefully suck up and discard working Silwet solution. Here again, you can use same capillary Pasteur pipette across multiple tubes. Add 1 mL of wIM using micropipette and close the lid. Perform embryo wash by gently inverting tubes for 10-15 times.

6.1.12. Using new capillary Pasteur pipette (can be used across multiple tubes), carefully suck up and

discard wIM solution. Add 1 mL each of Agro-suspension prepared in stepXX, and mix them by gently inverting tubes for 20 times. Lay tubes with mixture under the dark condition for 5 min.

6.1.13. During 5 min. of inoculation, pick up CoCult plate and label them accordingly (Experimental ID, host inbred, name or ID of construct and inoculation date).

Pour inoculated mixture (embryos and Agro-suspension altogether) to the corresponding CoCult plate. In the case that embryos remain in the test tubes, pick up and transfer them using knifes. Shake CoCult plate horizontally to draw a circle, now embryos will be gathered to the center.

6.1.14. Suck up Agro-suspension from CoCult plate using capillary Pasteur pipette and collect them to new empty 2 mL test tube (this suspension will be used later for embryo semi-submerging). Also remove Agro-suspension as much as possible around the embryos, as it makes following embryo flipping step easier. In these steps, you have to change capillary Pasteur pipette for every construct to avoid cross contamination.

Using scalpel blades, flip embryos to make them the flat-side (the side possesses shoot and root primordia) down and round-side (the side of scutellum) up. Also distribute the embryos widely on CoCult plate to not make them stacking with each other. Carefully check that all the embryos have been flipped and processed accordingly, as the embryos with wrong direction will have extremely low ability of callus induction.

6.1.15. Using 1000 µL micropipettes, transfer and drop 200-400 µL of Agro-suspension (collected at stepXX) on the flipped embryos to make them semi-submerged. The amount may depend on embryo size and numbers. Refer supplementary figure 2 for the degree of submerging. Seal dishes with parafilm and incubate CoCult plates at 22 °C for 3 overnights.

6.2. Embryo transfer to Resting medium: phase Resting (Day 4)

- Tools

2x scalpel blades (with holders, sterilized)
Surgical tape 12.5 mm width (to seal plates)

- Solutions and medium

Resting plates (warmed at room temperature in the dark before use. ~50 embryos per plate)

- Equipment and devices

Binocular microscopy (surface sterilize and placing under the sterile hood)
Flaming device together with a 70% ethanol bottle (placing under the sterile hood)
Incubation chamber set at 28 ºC and dark condition.

- Procedure and tips

From CoCult plate, pick up and transfer the embryos to the Resting plates using scalpel blade.

After finishing arrangement on the resting plate, push embryos downwards to perform embedding using backside of scalpel blade. Refer Fig.1 and SP1.Fig. 4 for the degree of embedding and culturing density on the plate.

Seal plates with surgical tape (to allow efficient air exchange and humidity control, not recommended to use parafilm or other tight-sealers) and incubate embryos at 28 ºC.

6.3. Embryo/Callus transfer to Sel-1 medium: phase Sel-1 (Day 14)

- Tools

2x Scalpel blades (with holders, sterilized)
2x angled tweezers, 15 cm length (sterilized)
Surgical tape 12.5 mm width (to seal plates)

- Solutions and medium

Sel-1 plates (warmed at room temperature in the dark before use. ~40 embryos/callus per plate)

- Equipment and devices

Binocular microscopy (surface sterilized and placing under the sterile hood)
Flaming device together with a 70% ethanol bottle (placing under the sterile hood)
Incubation chamber set at 28 ºC and dark condition.

- Procedure and tips

From Resting plates, pick up embryos which are forming embryonic callus on the scutellum region. Check the other side if the shoot primordium is elongating and cut shoot structure if there is. Embryos having no sign of callus induction (having flat and smooth structure of scutellum) can be discarded.

Transfer all callus positive embryos (regardless the degree of callus growth) to the Sel-1 plate. Using angled tweezers, perform embryo/callus embedding. Particularly in this stage, embed original embryo part into medium and let the callus region attached to the medium surface. Also refer Fig. 1A and SP1-Fig. 5 for the degree of embedding and culturing density on the plate.

Seal plates with surgical tape (you can reuse surgical tape used in previous phase, if it is clean enough) and incubate embryos at 28 ºC.

6.4. Callus transfer to Sel-2 medium: phase Sel-2 (Day 28)

- Tools

2x Scalpel blades (with holders, sterilized)
2x angled tweezers, 15 cm length (sterilized)
Surgical tape 12.5 mm width (to seal plates)

- Solutions and medium

Sel-2 plates (warmed at room temperature in the dark before use. ~ 25 calli per plate)

- Equipment and devices

Binocular microscopy (surface sterilized and placing under the sterile hood)
Flaming device together with a 70% ethanol bottle (placing under the sterile hood)
Incubation chamber set at 28 ºC and dark condition.

- Procedures and tips

From Sel-1 plates, sort and pick up calli which experienced active proliferation. Calli showing highly watery texture or brownish colors overall can be discarded in this step.

After sorting, remove original embryo part of positive callus (is usually turned into brown-colored and having hard texture) using scalpel blades. If the callus has experienced extensive proliferation already, like being callus chank bigger than 10 mm diameter, perform callus splitting into half size.
(Tips) To do this cutting works, use petri-dish surface exposed by removing medium, rather than performing cutting on the unstable medium surface. If you are right-handing person, holding tweezers in right hand and a scalpel blade in left hand may help to perform efficient work. (These tips are same for later phases).

Transfer and arrange treated callus into Sel-2 plates, with directing cutting surface down as it's contacting to medium surface. Perform callus embedding to the degree that the callus equator circumference is slightly embedded in the medium. Also refer Fig. 1A and SP1-Fig. 6 for the degree of embedding and culturing density on the plate.

Seal plates with surgical tape (you can reuse surgical tape used in previous phase, if it is clean enough) and incubate calli at 28 ºC.

6.5. Callus transfer to Sel-3 medium: phase Sel-3 (Day 42)

- Tools

2x Scalpel blades (with holders, sterilized)
2x angled tweezers, 15 cm length (sterilized)
Surgical tape 12.5 mm width (to seal plates)

- Solutions and medium

Sel-3 plates (warmed at room temperature in the dark before use. ~25 calli per plate)

- Equipment and devices

Binocular microscopy (surface sterilized and placing under the sterile hood)
Flaming device together with a 70% ethanol bottle (placing under the sterile hood)
Incubation chamber set at 28 ºC and dark condition.

- Procedures and tips

From Sel-2 plates, sort and pick up calli which experienced active proliferation. Again, calli showing highly watery texture or brownish colors overall can be discarded in this step. Typically, most of the calli in this phase show mixed morphology which possess negative sectors (characterized by brownish colors with extremely hard texture) and positive-looking sector keeping white and folding-rich structure. So that, usually you will perform the trimming of callus, to remove negative sectors. as well as callus splitting of positive-looking sectors into appropriate size if it's necessary (into pieces smaller than 5 mm diameter, also see Result section regarding "regeneration through splitting" concept in main text). You may also find calli those already start to show part of committed resistance callus (CoReCa) morphology (see Result section of main text), and keep those calli accordingly.

Transfer and arrange treated callus into Sel-3 plates, again with directing cutting surface down as it's contacting to medium surface. Perform callus embedding to the similar degree as Sel-2 phase. Also refer Fig. 1A and SP1-Fig. 7 for the degree of embedding and culturing density on the plate.

Seal plates with surgical tape (you can reuse surgical tape used in previous phase, if it is clean enough) and incubate calli at 28 ºC.

6.6. Callus transfer to Reg1 medium: phase Reg1-1 (Day 56)

- Tools

2x Scalpel blades (with holders, sterilized)
2x angled tweezers, 15 cm length (sterilized)
Surgical tape 12.5 mm width (to seal plates)

- Solutions and medium

Reg1 plates (warmed at room temperature in the dark before use. ~25 calli per plate)

- Equipment and devices

Binocular microscopy (surface sterilized and placing under the sterile hood)
Flaming device together with a 70% ethanol bottle (placing under the sterile hood)
Incubation chamber set at 26 ºC and dark condition.

- Procedures and tips

In this phase, the most important handling is to perform morphological selection of positive CoReCa. As described in Result section, CoReCa morphology is not only characterized by visuals but also by textures, so that examine calli by actually touching them with tweezers and scalpel blades. On the other hand, size control of callus by spitting is another important concept for the following regeneration phases, therefore, you may examine callus texture by actual splitting in some cases. In particular, CoReCa-looking callus having easy-to-split feature (but not hard-to-split) is most probable positive callus capable to regenerate positive plantlets in the following phases.

Smaller callus sector with white-colored but having extremely condensed tissue morphology (CoReCa is typically rather loose tissue morphology) is mostly negative and to be discarded. However, in the case you wonder if you should keep the callus or not, just transfer such a callus to the next phase and examine in Reg1-1.

Transfer and arrange treated callus into Reg1 plates. From this phase, no need to perform callus embedding. Also refer Fig. 1A and SP1-Fig. 8 for the culturing density on the plate.

Seal plates with surgical tape (you can reuse surgical tape used in previous phase, if it is clean enough) and incubate calli at 26 ºC.

6.7. Callus transfer to Reg1 medium: phase Reg1-2 (Day 66)

- Tools

2x Scalpel blades (with holders, sterilized)
2x angled tweezers, 15 cm length (sterilized)
Surgical tape 12.5 mm width (to seal plates)

- Solutions and medium

Reg1 plates (warmed at room temperature in the dark before use. ~25 calli per plate)

- Equipment and devices

Binocular microscopy (surface sterilized and placing under the sterile hood)
Flaming device together with a 70% ethanol bottle (placing under the sterile hood)
Incubation chamber set at 26 ºC and dark condition.

- Procedures and tips

At this timing, typically CoReCa-derived and other possible positive callus starts to form root primordium (is characterized by root hairs) as well as shoot primordium (is characterized by a bulb-like structure). In some cases, positive callus may continue to proliferate rather than start to differentiate meristems. In either case, perform size control of callus by splitting and transfer them to the fresh Reg1 plates. Also, refer SP1-Fig. 9 for the culturing density on the plate.
Calli having no sign of proliferation or differentiation can be discarded in this step. Also, trim and discard negative sectors showing dark-brown or black color, or degenerating morphologies.

Seal plates with surgical tape (you can reuse surgical tape used in previous phase, if it is clean enough) and incubate calli at 26 ºC.

6.8. Callus transfer to Reg2 medium: phase Reg2p (Day 76)

- Tools

2x Scalpel blades (with holders, sterilized)
2x angled tweezers, 15 cm length (sterilized)
Surgical tape 12.5 mm width (to seal plates)

- Solutions and medium

Reg2 plates (prepared in 20 mm height petri-dishes, warmed at room temperature before use. ~20 calli per plate)

- Equipment and devices

Binocular microscopy (surface sterilized and placing under the sterile hood)
Flaming device together with a 70% ethanol bottle (placing under the sterile hood)
Incubation chamber set at 26 ºC with grow lights (long-day or continuous)

- Procedures and tips

The aim of Reg2p phase is to shape regenerating shoots growing upwards, but not let them elongate horizontal direction due to limited aerial space. This is the reason why you are supposed to use higher petri dishes for Reg2p medium.

After Reg1-2 phase, typical CoReCa-derived callus elongate root structure quite extensively and regenerate some shoot structures. Intact shoot structure and shoot containing/forming callus are the preferred targets for the following culture phase. To visualize potential shoots, expose Reg1-2 cultures under the light for 2-3 hours or an overnight prior to perform callus sorting/splitting/trimming, so that shoot structures will be turned into yellowish or pale-green color thanks to the chloroplast activation. Shoot meristematic tissues usually locate/attach on the surface of callus, however, sometimes callus may have runner-like stem structure and generate shoot on the top of it. In this case, find node-like structure and meristematic tissues can locate there. No need to have the sign of root formation yet, as they (= adventitious roots) will be formed in later phases.

On the other hand, extensive root structures often prevent to perform efficient tissue handling and may annoy you. Cut roots into short length (~5 mm), and most of the cases, new root primordia will be extensively formed nearby again in the following phases.

Perform callus splitting according to the regeneration through splitting concept. In case that two or more distinguishable shoots are formed with enough distances in a single callus, try to split into differential units. However, let them bundle together in the case they have a danger to be injured due to splitting.
You may also need to consider the splitting strategy according to the actual application. For CRISPR/Cas9 mutagenesis, indeed progressive splitting may be preferred to isolate independent editing events. For the reporters, on the other hand, keeping two or multiple shoots bundled may be a preferred option as they mostly share same T-DNA insertion event.

Transfer treated calli to the Reg2p plates and perform embedding. The optimal degree of embedding is that the shoot meristematic region is contacting to the medium surface, so that regenerating shoot can form plantlet unit structure (shoot plus roots) in the following stage.

Seal plates with surgical tape (you can reuse surgical tape used in previous phase, if it is clean enough) and incubate calli at 26 °C with light.

Also refer SP1-Fig. 10 for the callus handling guide in this phase.

6.9. Shoot/Callus transfer to Reg2 bottles: phase Reg2b (Day 83)

- Tools

2x Scalpel blades (with holders, sterilized)
2x angled tweezers, 15 cm length (sterilized)
2x straight tweezers, 20 cm length (sterilized)

- Solutions and media

Reg2 bottles (prepared in 500 mL glass jars, warmed at room temperature before use. ~15 calli/shoots per bottle)

- Equipment and devices

Binocular microscopy (surface sterilized and placing under the sterile hood)
Flaming device together with a 70% ethanol bottle (placing under the sterile hood)
Incubation chamber set at 26 ºC with grow lights (long-day or continuous)

- Procedures and tips

The main purpose of Reg2b phase is to establish robust plantlet structure capable to be transferred to soil and subjected to acclimatization. Now you can easily distinguish shoots and other structures thanks to the deep-green colored leaves. Same procedures from Reg2p phase can be applicable for shoots/calli handling. You may find some bushy regenerants which possess incomplete leaf sheath and leaf blade structure without appropriate folding. Such a bushy leaf callus is still good source to establish a plantlet (mostly this will form multiple shoots), therefore keep and transfer to the next phase just leaving it as a chank.

Using longer straight tweezers, transfer and insert treated plantlet deep inside the medium. As long as a part of emerging leaves are exposed to aerial area, the plantlet will grow vigorously.

Close and lock the lid of jars (no need to have sealing with surgical tape) and incubate plantlet at 26 ºC with light.

It usually takes 7-10 days to go to next acclimatization step, depending on the shoot status in the beginning. The morphological criterion is that the timing when leaves touch the lid of glass jar (10 cm height). Do not culture plantlets extremely longer in this phase as high humidity (due to active transpiration of plantlets) and excessive amount of water drop may degenerate emerging leaves, and

this affect survival rate of plantlets during acclimatization. In particular, plantlets derived from B104 are more sensitive for this humidity stress, so that you can shorten this phase to 5-7 days regardless the resultant plantlet size.

When you are planning to make a cross with T0 plants, the beginning of Reg2b phase is the timing that the seeds of crossing partners (in case of sharing same inbred background) to be sown. Preparing other rounds in ± 4-7 days is highly recommended to make crosses surely possible.

Also refer SP1-Fig. 11 for the callus/plantlet handling guide in this phase.

6.10. Plantlet transfer to soil trays or pots: phase Acclimatization (Day 93)

- Procedures and tips

Using hands (with gloves), carefully pick plantlets up from glass jars, remove excessive amount of medium from the root system (leaving little amount is acceptable) and transfer them to the cell-trays or small pots filled with soil. Treat bushy regenerants as a chunk as described above. During the handling, collect treated plantlet on the tray with wet paper towels to prevent drying. After finishing this transplanting, immediately shower plantlets with enough amount of water. As excessive amount of water drop is disadvantageous during acclimatization, do not cover the trays or pots, rather, allow them to access to fresh air.

One of undesired developmental features often observed during acclimatization is that degenerated leaf is preventing the emergence of new leaves coming from inside and stimulate another stacking-degeneration cycle. Therefore, check plant morphology regularly during acclimatization (once per every second days), and solve such a situation by cutting stacking part of the emerging leaves (SP1-Fig. 12). It is the sign of completion of acclimatization when you see healthy, non-degenerative and well-opened leaves are constantly emerging from inside.

In case you have treated some plantlets as multi-shoot structure, choose and leave well-grown one or two shoots and remove others before transplanting to big pots. In our trials, plantlets having more than three shoots did not experience better growth anymore, probably due to space and nutrient competition among shoots, and twin-shoot were acceptable.

7. Figures and figure legends for Supporting Experimental Procedure 1 (SP1-Figs)

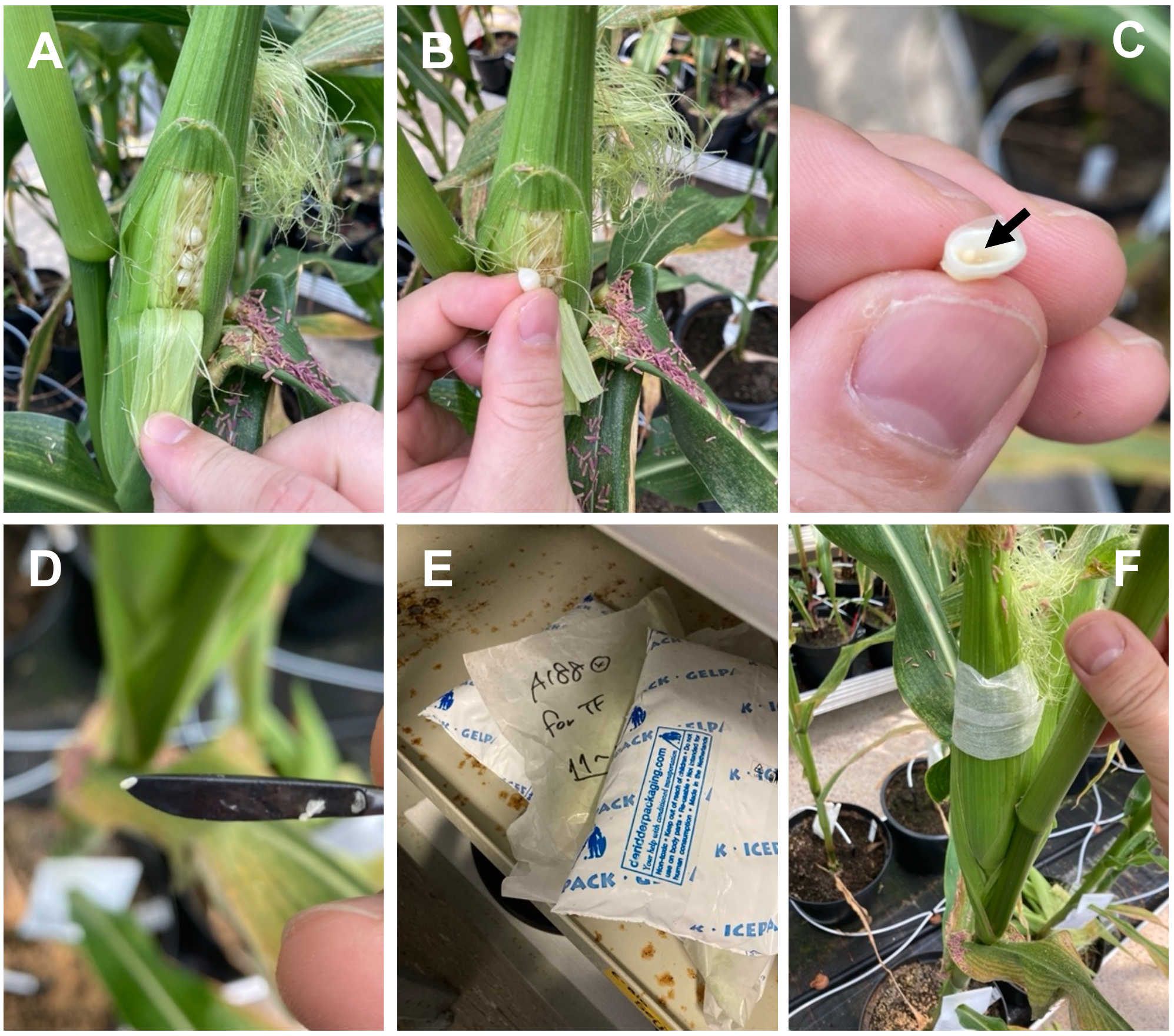

SP1-Fig. 1 Harvesting maize cobs and immature embryos suitable for transformation.

A. Accessing developing kernel by opening a window on bracts.

B. Picking up one kernel to test embryo development

C. Developing kernel after removal of endosperm, allow indicates immature embryo.

D. An immature embryo scooped from developing kernel, showing 1.3 mm vertical length.

E. Storing harvested cobs in between cooling gel pads.

F. A cob sealed with surgical tapes, in the case that the cob needs additional growth.

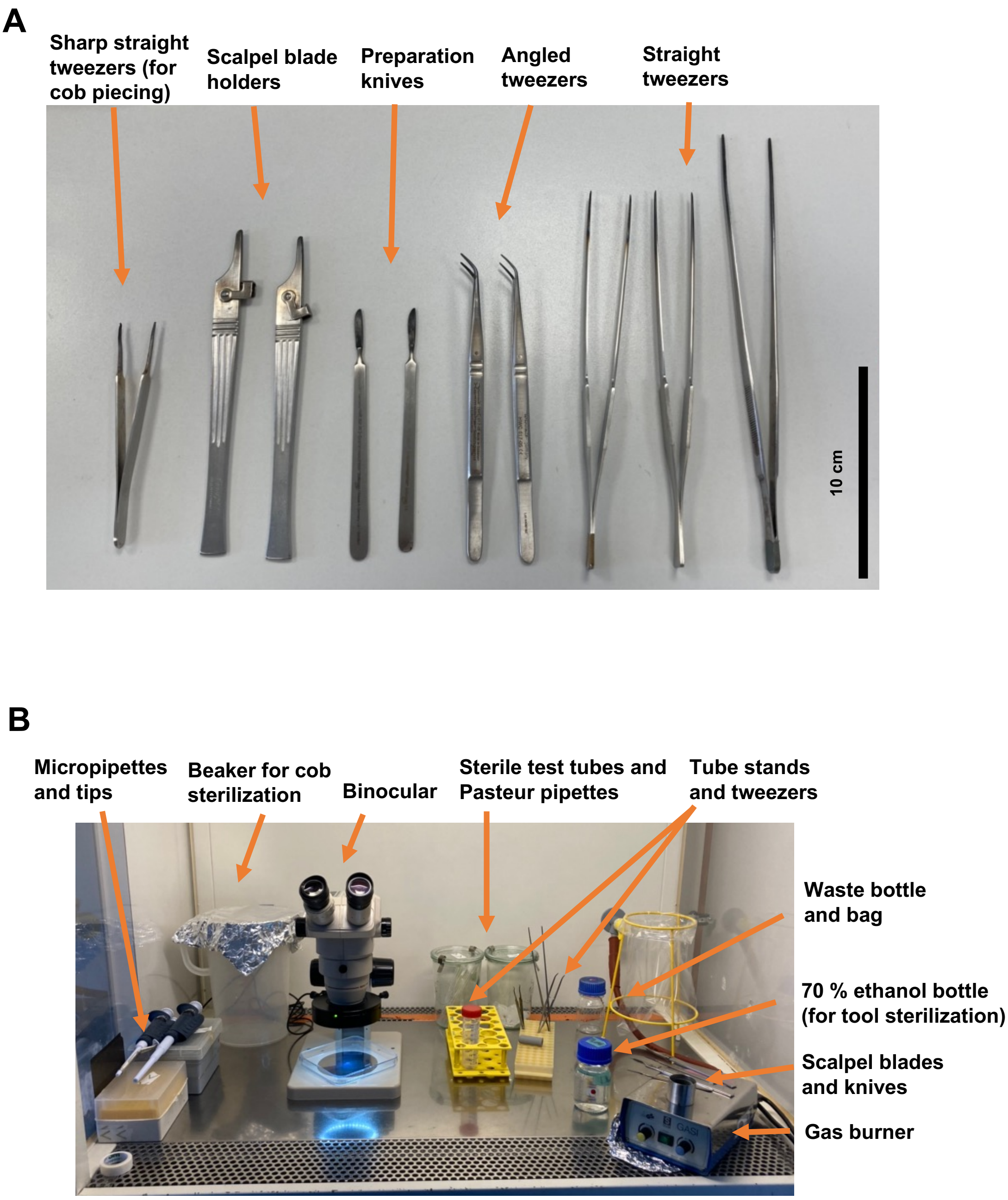

SP1-Fig. 2 Tools and bench set up for maize transformation.

A. Tools for tissue culture works of maize transformation. Note that having two sets of scalpel blade holder (with scalpel blade), knife and tweezers is beneficial to use them in a rotating manner during tissue culture works.

B. An example of workspace setting up under the sterile hood, for the day of embryo isolation and inoculation (Day 1).

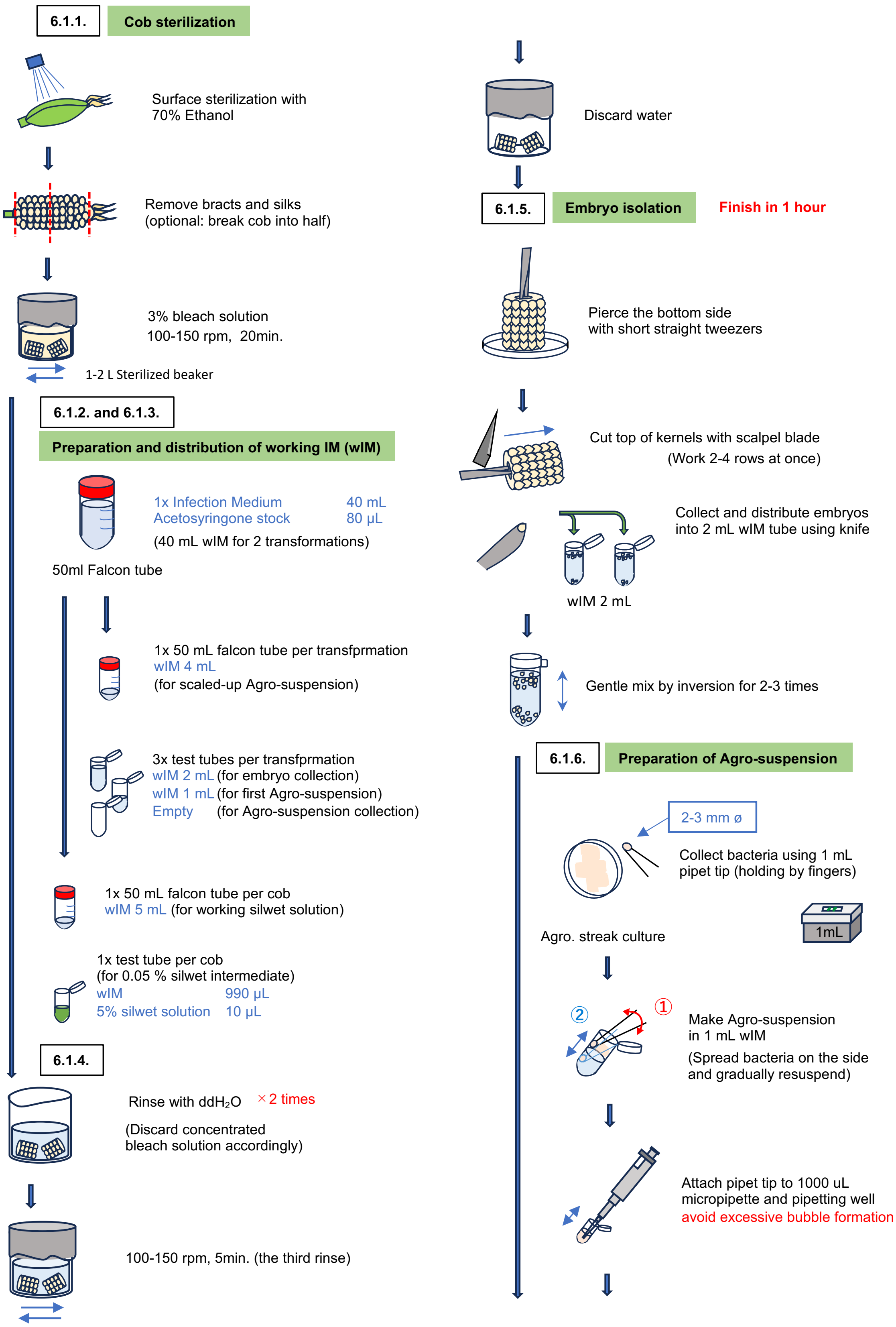

6.1.1.
Cob sterilization
Surface sterilization with 70% Ethanol
Remove bracts and silks (optional: break cob into half)
3% bleach solution
100-150 rpm, 20min.
1-2 L Sterilized beaker
6.1.2. and 6.1.3.
Preparation and distribution of working IM (wIM)
1x Infection Medium 40 mL
Acetosyringone stock 80 µL
(40 mL wIM for 2 transformations)
50ml Falcon tube
1x 50 mL falcon tube per transfprmation
wIM 4 mL
(for scaled-up Agro-suspension)
3x test tubes per transfprmation
wIM 2 mL (for embryo collection)
wIM 1 mL (for first Agro-suspension)
Empty (for Agro-suspension collection)
1x 50 mL falcon tube per cob
wIM 5 mL (for working silwet solution)
1x test tube per cob
(for 0.05 % silwet intermediate)
wIM 990 µL
5% silwet solution 10 µL
6.1.4.
Rinse with ddH$_2$O ×2 times
(Discard concentrated bleach solution accordingly)
100-150 rpm, 5min. (the third rinse)
Discard water
6.1.5.
Embryo isolation
Finish in 1 hour
Pierce the bottom side with short straight tweezers
Cut top of kernels with scalpel blade (Work 2-4 rows at once)
Collect and distribute embryos into 2 mL wIM tube using knife
wIM 2 mL
Gentle mix by inversion for 2-3 times
6.1.6.
Preparation of Agro-suspension
2-3 mm ø
Collect bacteria using 1 mL pipet tip (holding by fingers)
Agro. streak culture
1mL
①
②
Make Agro-suspension in 1 mL wIM
(Spread bacteria on the side and gradually resuspend)
Attach pipet tip to 1000 uL micropipette and pipetting well
avoid excessive bubble formation

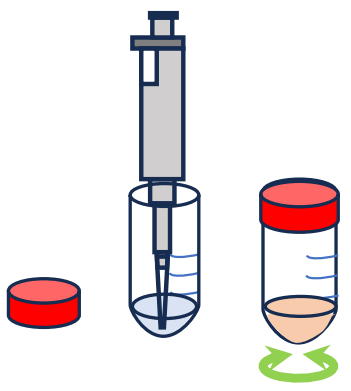

Add Agro solution into 4 mL wIM
Gentle mix by shaking horizontally

wIM 4 mL + first Agro-suspension 1 mL

6.1.7.

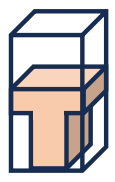

Take 1 mL scaled-up Agro-suspension to measure OD_{600}

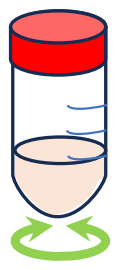

Adjust to **OD_{600}=0.4** with wIM

(Equation) **Y = 10*X - 4**

X: Measured OD_{600} value
Y: Vol. wIM to add (mL)

6.1.8. **Preparation of working silwet solution**

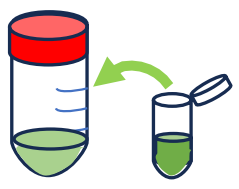

Add 0.05 % silwet intermediate to 5 mL wIM to make working silwet solution

Amount will differ according to applied Silwet concentration

wIM 5 mL + 0.05 % silwet intermediate

6.1.9. **Preparation of capillary Pasteur pipettes**

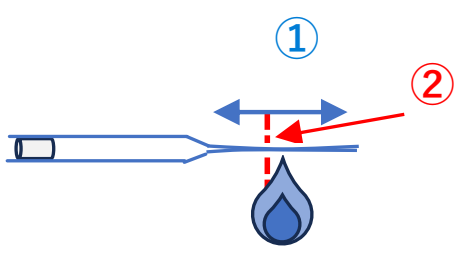

Stretch glass Pasteur pipettes
And break at appropriate thinness

Prepare No. of transformation plus 1

6.1.10. **Flash silwet treatment**

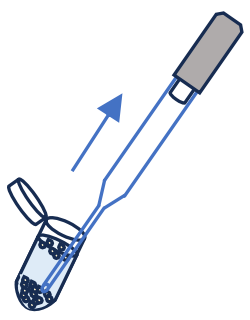

Discard solution

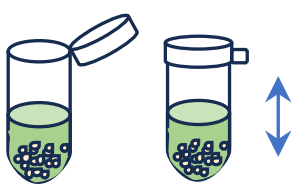

Add 1 mL working Silvet solution
Gentle mix by inversion for 2-3 times

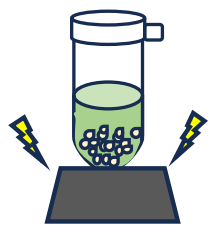

Flash vortex 1 sec. for 10 times
(with inverting 1-2 times every interval)

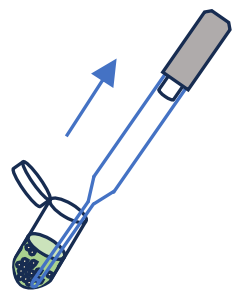

Discard solution

6.1.11. **Embryo wash**

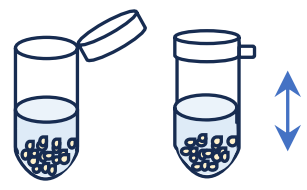

Add 1 mL wIM
Gentle mix by inversion for 2-3 times

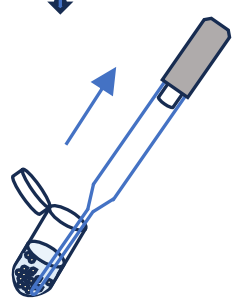

Discard solution using a new pipet

6.1.12. **Inoculation**

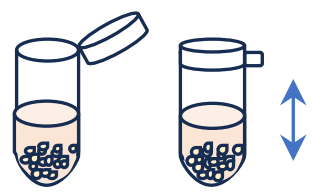

Add 1 mL Agro-suspension (OD_{600}= 0.4)
Gentle mix by inversion for 20 times

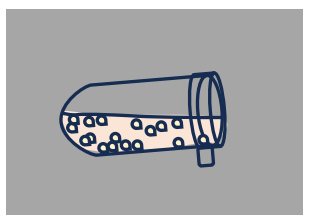

Incubate at room temperature, 5min., in the dark

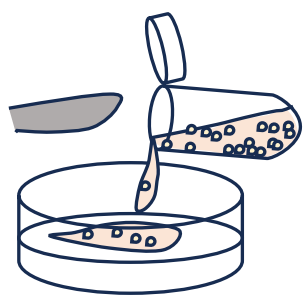

6.1.13.

Pour embryos and Agro-suspension onto CoCult plate
(use knife to scoop up and transfer remaining embryos)

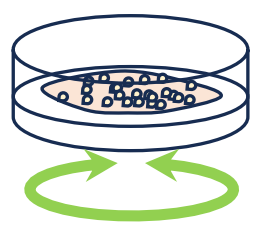

Shake plate horizontally to gather embryos to the center

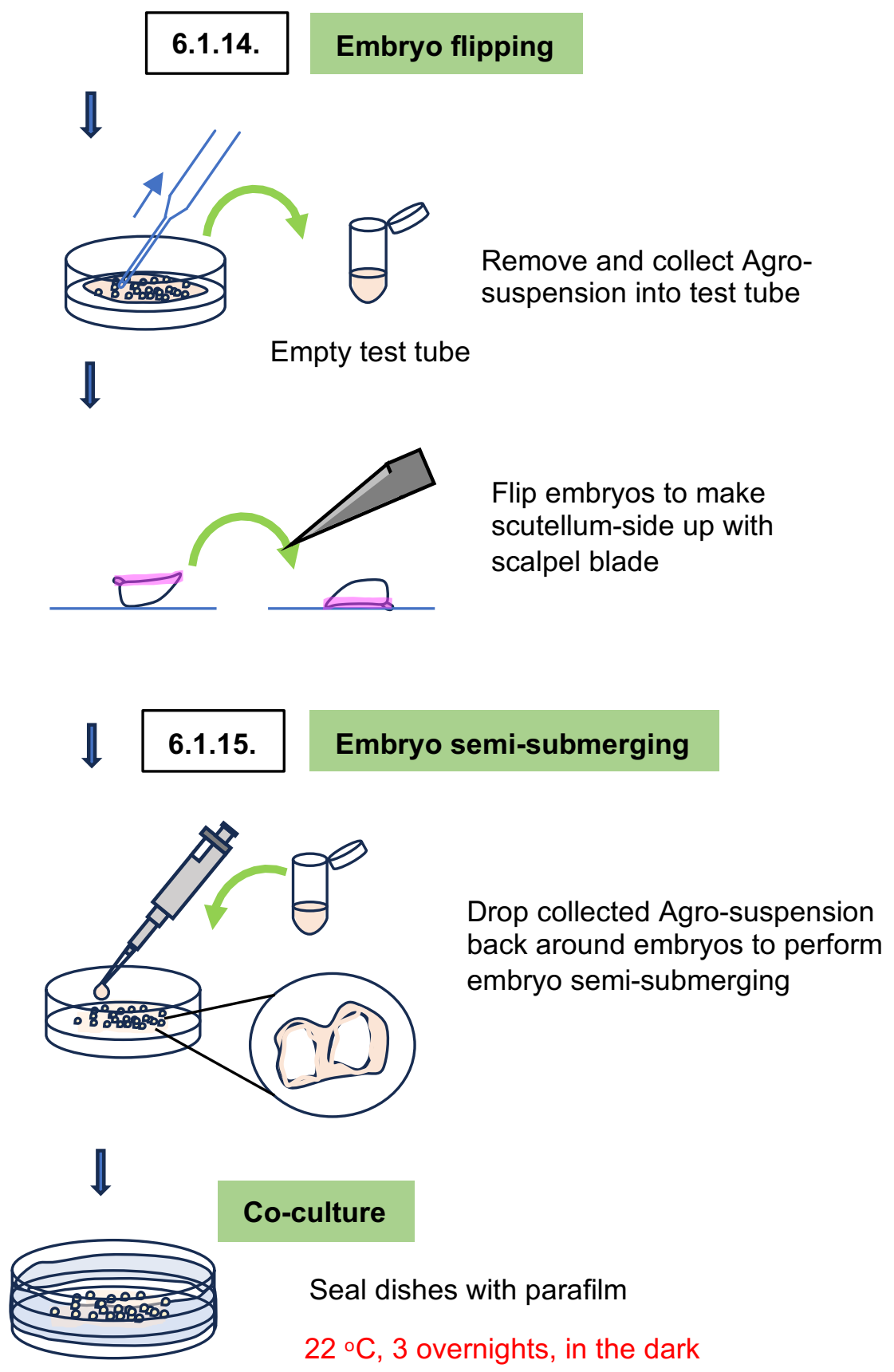

SP1-Fig. 3 An illustrated workflow for embryo isolation and inoculation.

The workflow is shown in two columns (left to right) in each page. Numbers shown in the rectangle outlines are corresponding to the steps described in the text.

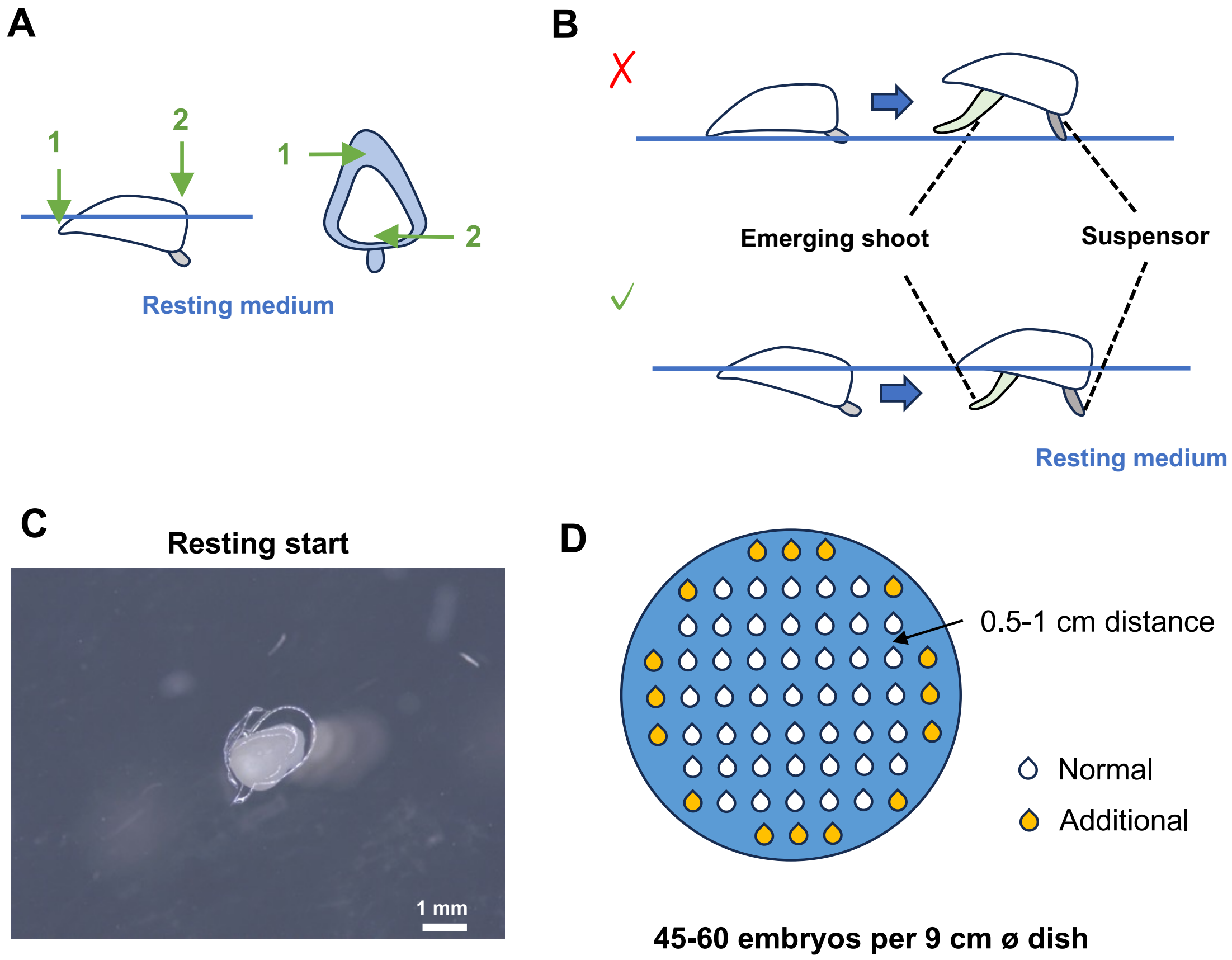

SP1-Fig. 4 Schematic illustrations and examples of tissue handling for Resting phase.

A. An illustration showing the optimal degree of embedding for Resting phase. Allows with numbers represent the points to push down into the medium.

B. An illustration showing expected embryo growth without (upper, undesirable) and with (lower, preferrable) embedding.

C. A picture showing the optimal degree of embedding for Resting phase.

D. An illustration representing the optimal density of embryo culture during Resting phase.

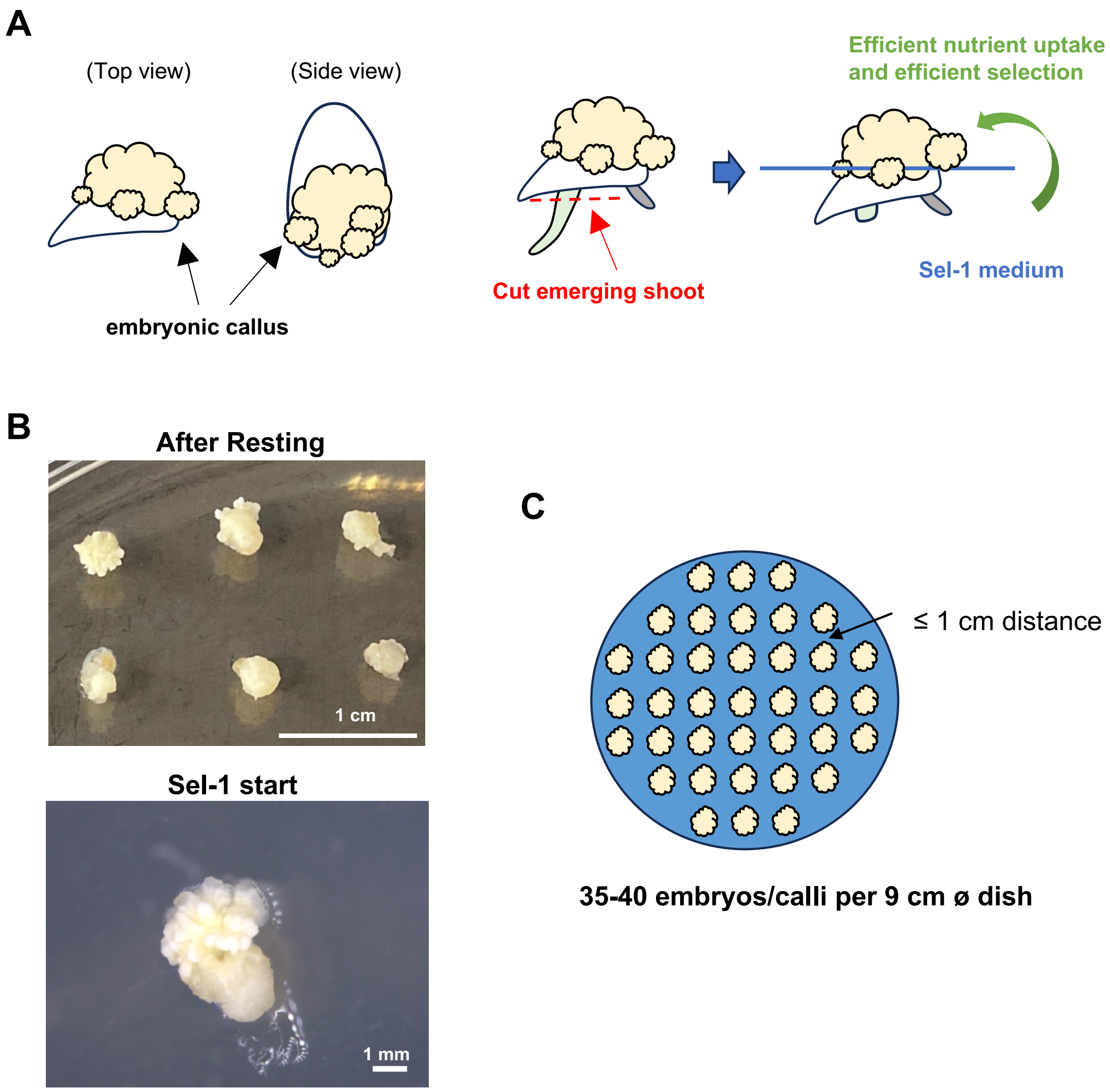

SP1-Fig. 5 Schematic illustrations and examples of tissue handling for Sel-1 phase.

A. An illustration showing typical callus growth after Resting phase, guidance for shoot removal and optimal degree of embedding for Sel-1 phase.

B. Pictures showing typical callus growth after Resting phase and the optimal degree of embedding for Sel-1 phase.

C. An illustration representing the optimal density of embryo culture during Sel-1 phase.

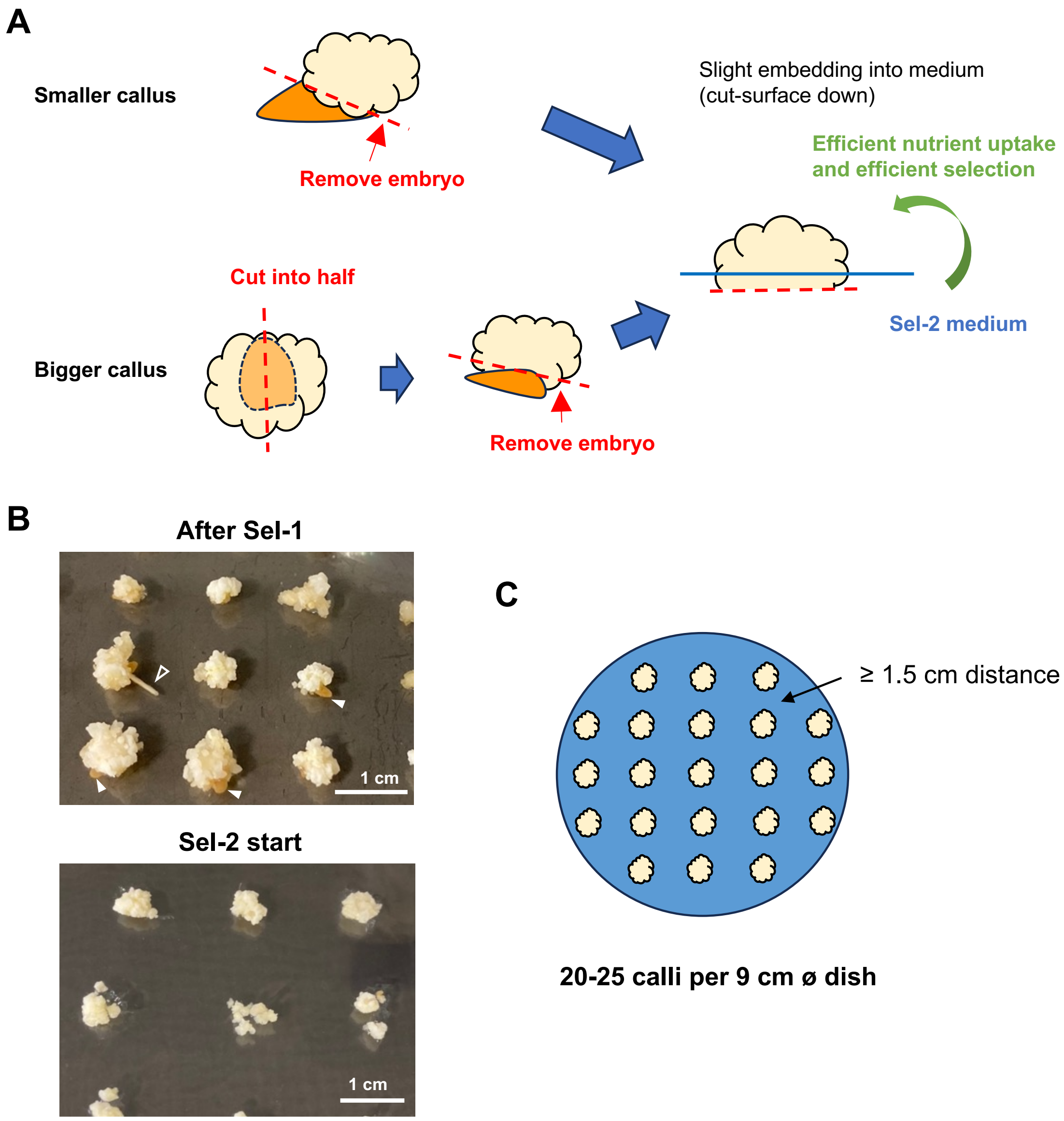

SP1-Fig. 6 Schematic illustrations and examples of tissue handling for Sel-2 phase.

A. An illustration showing typical callus growth after Sel-1 phase, guidance for embryo removal and optimal degree of embedding for Sel-2 phase.

B. Pictures showing typical callus growth after Sel-1 phase and selected and trimmed calli for Sel-2 phase.

C. An illustration representing the optimal density of embryo culture during Sel-2 phase.

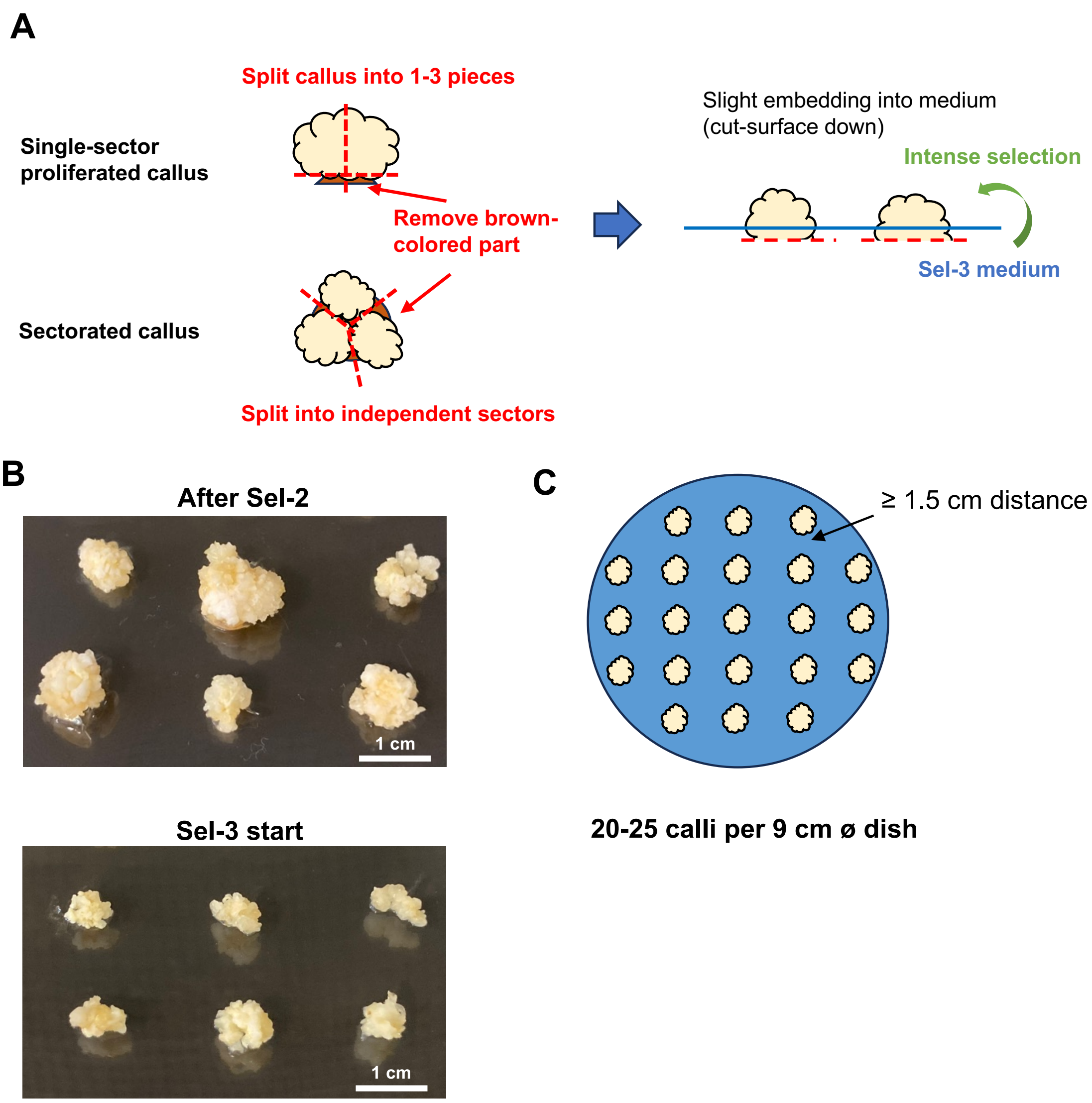

SP1-Fig. 7 Schematic illustrations and examples of callus handling for Sel-3 phase.

A. An illustration showing typical callus growth after Sel-2 phase, guidance for callus trimming and optimal degree of embedding for Sel-3 phase.

B. Pictures showing typical callus growth after Sel-2 phase and selected and trimmed calli for Sel-3 phase.

C. An illustration representing the optimal density of embryo culture during Sel-3 phase.

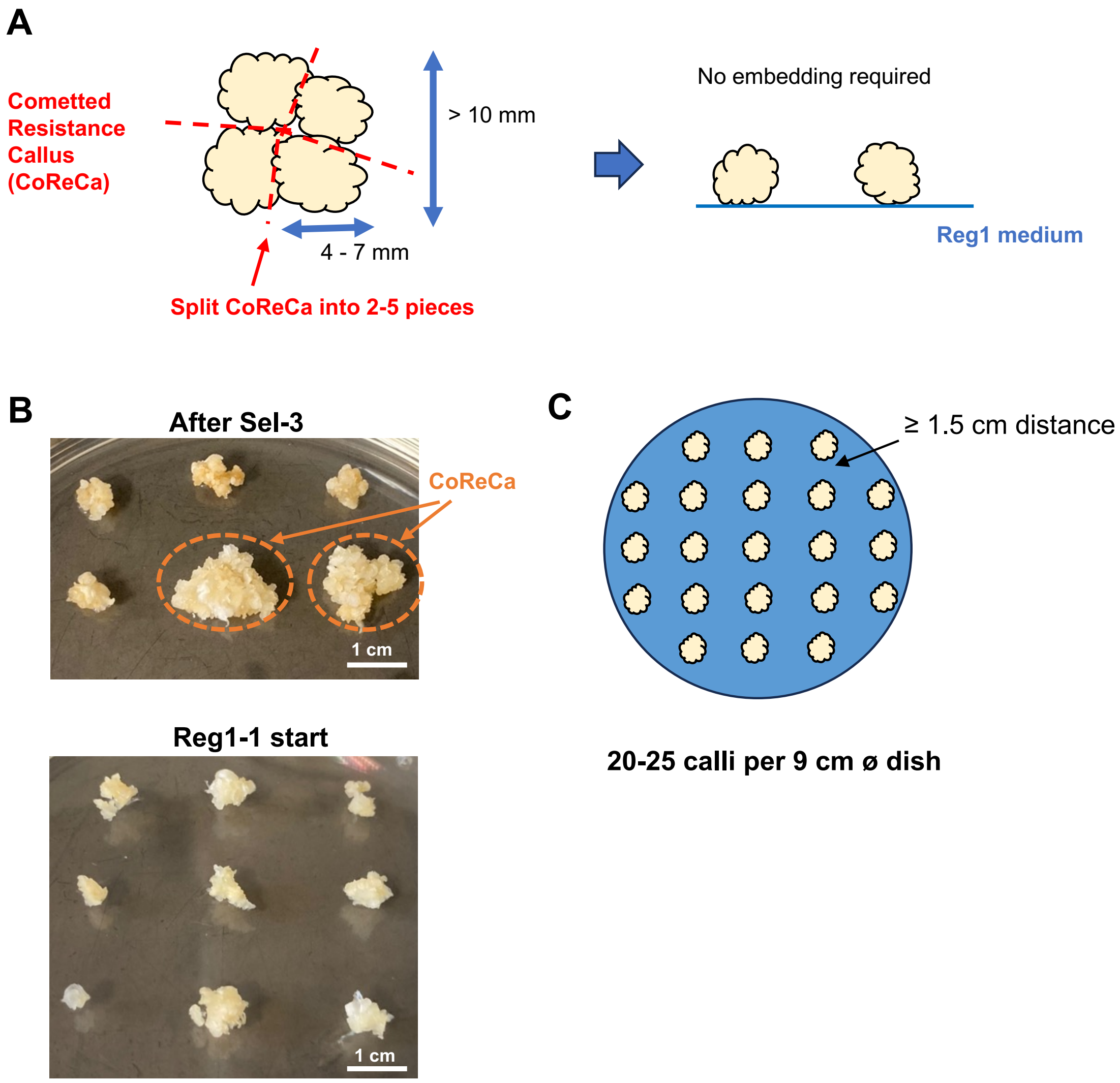

SP1-Fig. 8 Schematic illustrations and examples of callus handling for Reg1-1 phase.

A. An illustration showing typical callus growth after Sel-2 phase, guidance for callus splitting and optimal tissue culturing for Reg1-1 phase.

B. Pictures showing typical callus growth after Sel-3 phase and appearance of CoReCa, and selected and split calli for Reg1-1 phase.

C. An illustration representing the optimal density of embryo culture during Reg1-1 phase.

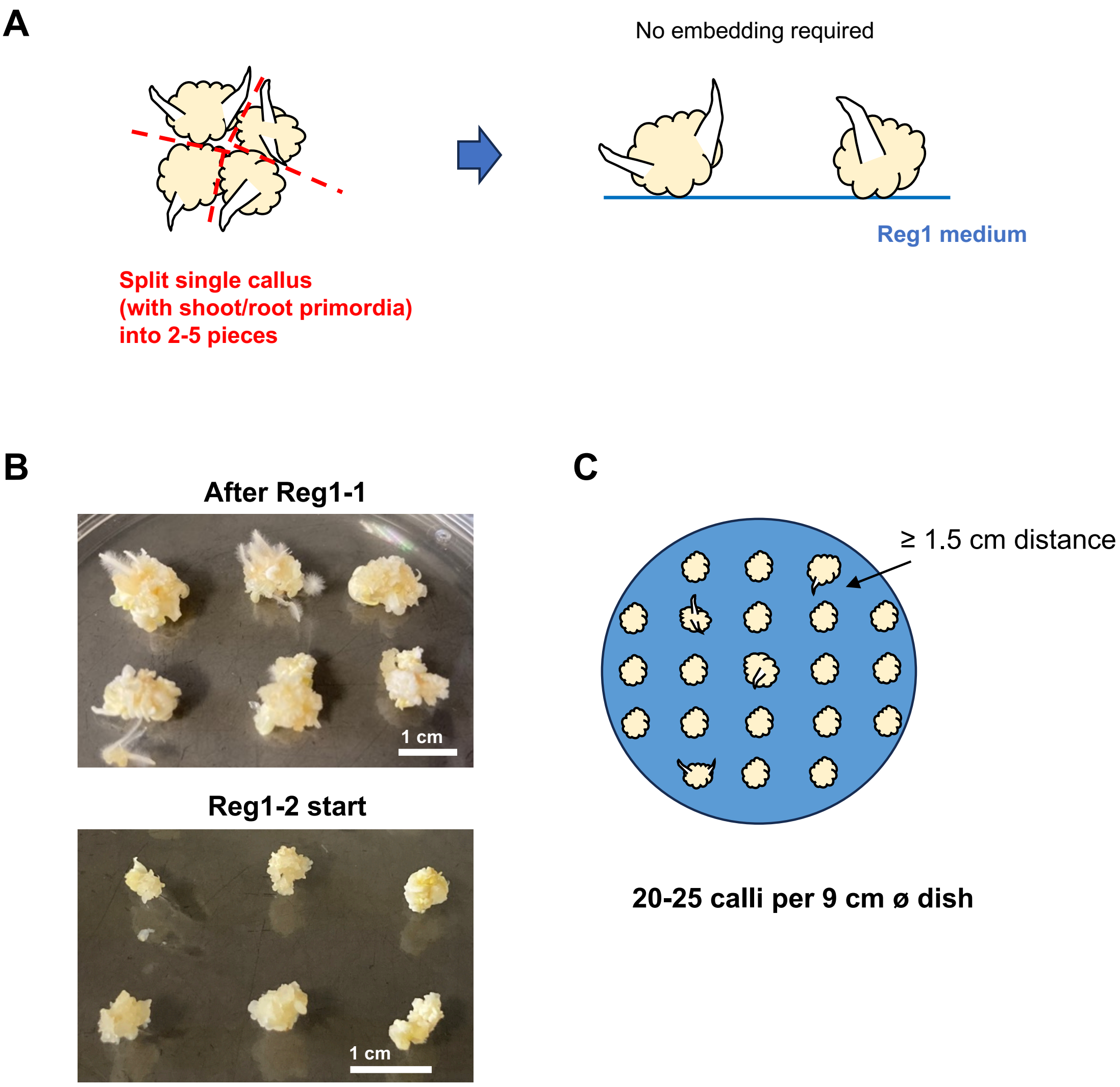

SP1-Fig. 9 Schematic illustrations and examples of callus handling for Reg1-2 phase.

A. An illustration showing typical callus growth after Reg1-1 phase, guidance for callus splitting and optimal tissue culturing for Reg1-2 phase.

B. Pictures showing typical callus growth after Reg1-1 phase and selected and split calli for Reg1-2 phase.

C. An illustration representing the optimal density of embryo culture during Reg1-2 phase.

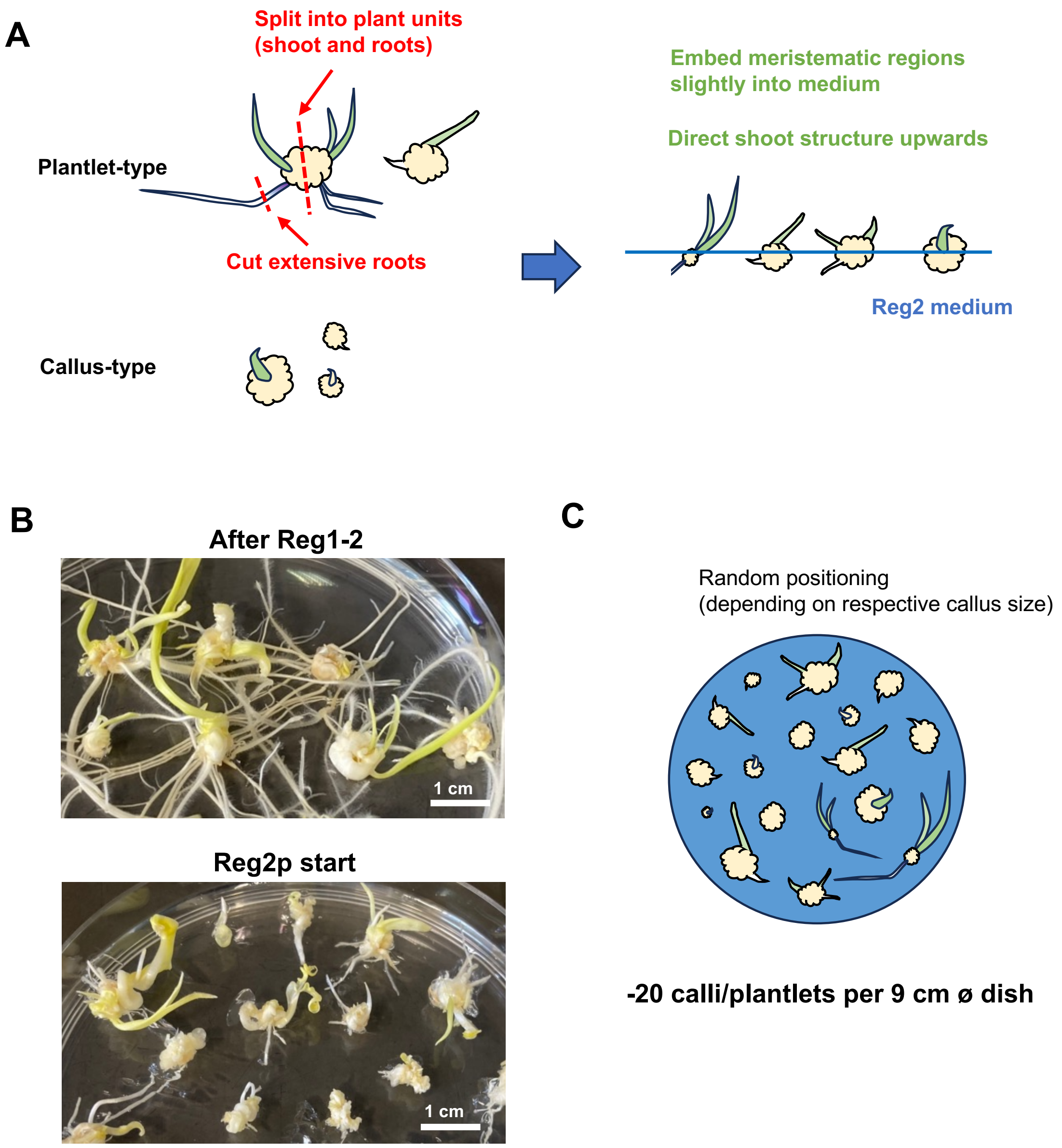

SP1-Fig. 10 Schematic illustrations and examples of tissue handling for Reg2p phase.

A. An illustration showing typical callus/plantlet growth after Reg1-2 phase, guidance for callus splitting and trimming and optimal tissue embedding for Reg2p phase.
B. Pictures showing typical callus growth after Reg1-2 phase and selected and split calli/plantlets for Reg2p phase.
C. An illustration representing the optimal density of embryo culture during Reg2p phase.

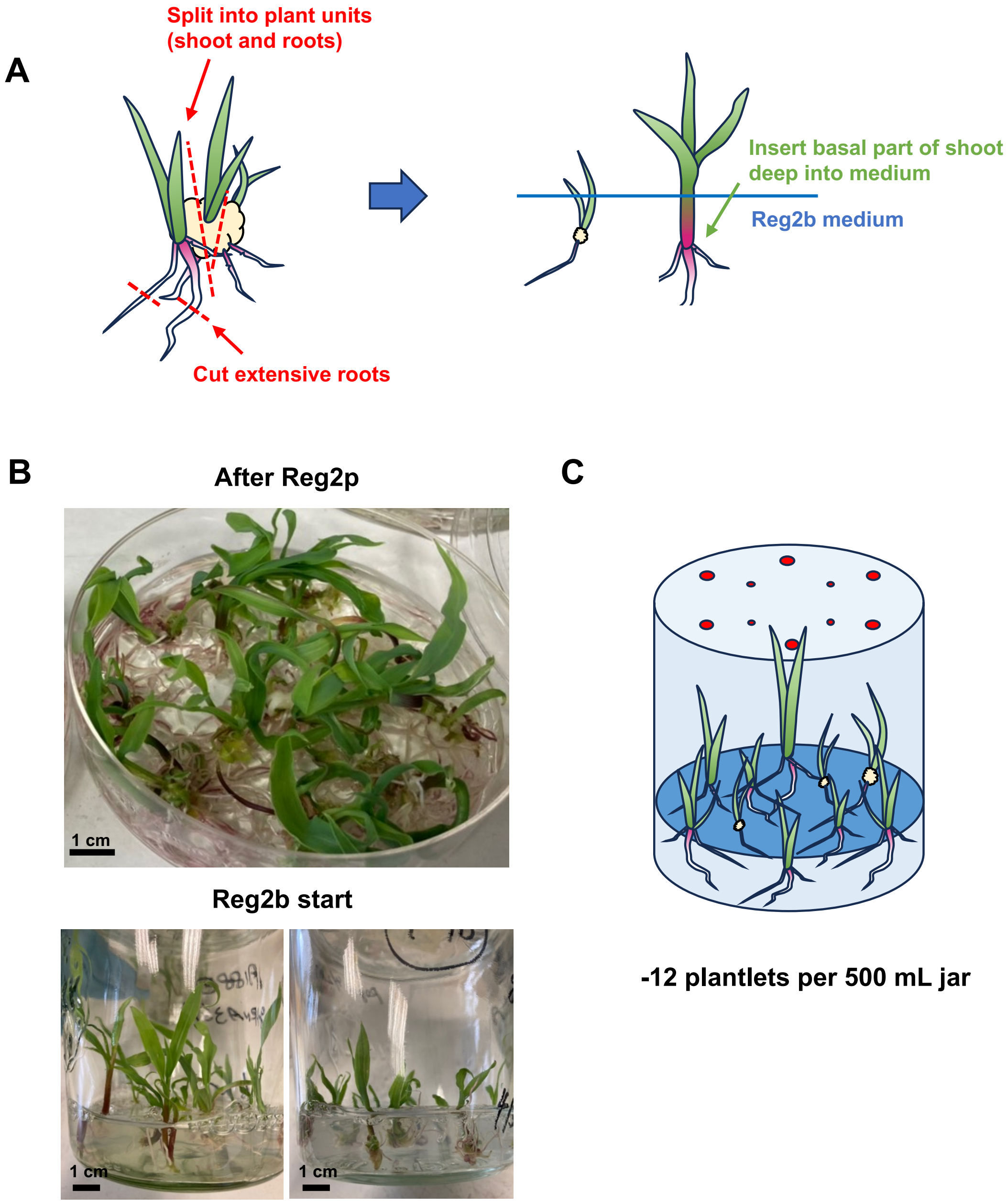

SP1-Fig. 11 Schematic illustrations and examples of tissue handling for Reg2b phase.

A. An illustration showing typical plantlet structure after Reg2p phase, guidance for tissue splitting and trimming and optimal tissue embedding for Reg2b phase.

B. Pictures showing typical callus/tisssue growth after Reg2p phase and selected plantlets for Reg2b phase.

C. An illustration representing the optimal density of embryo culture during Reg2b phase.

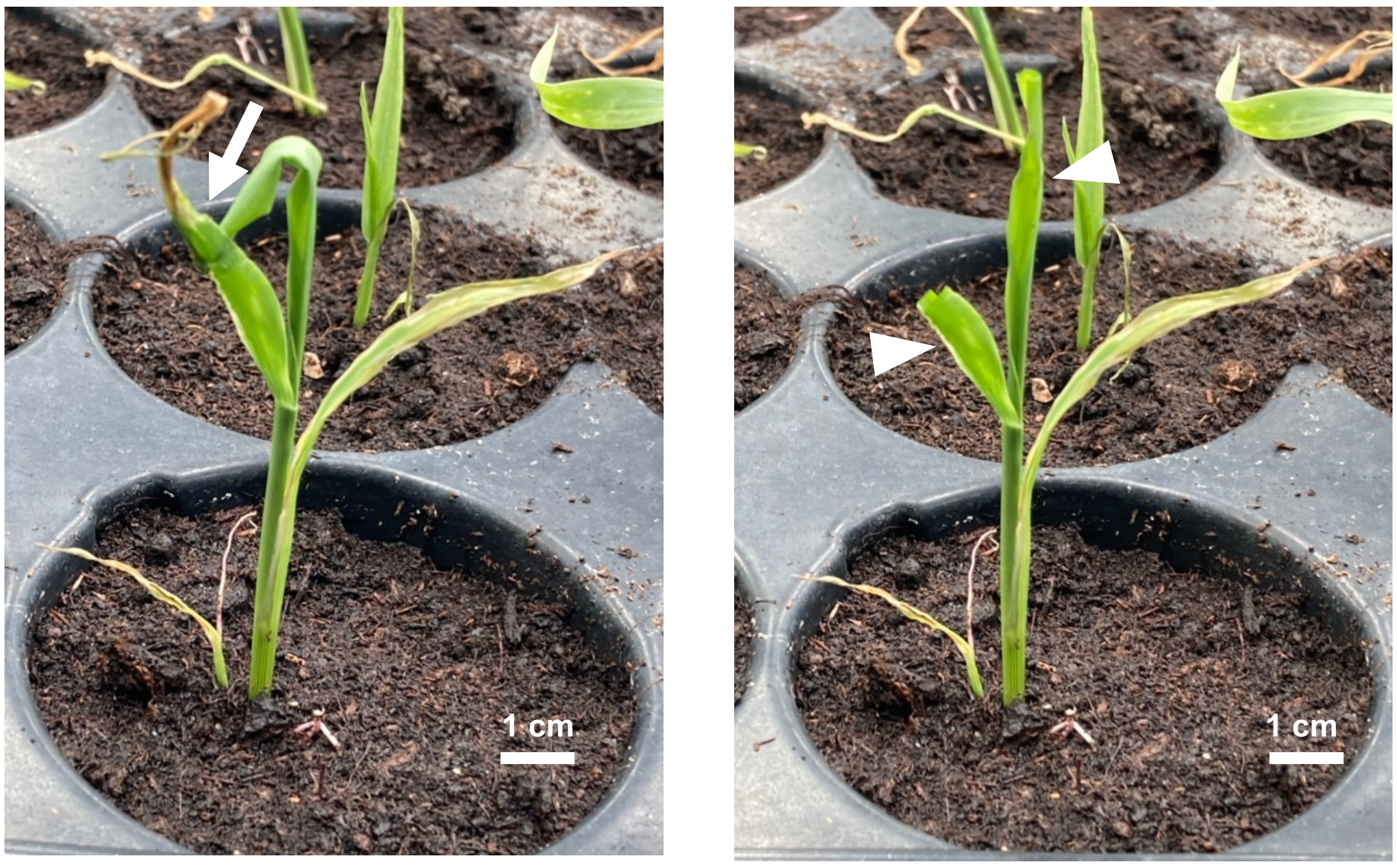

SP1-Fig. 12 Stacking of emerging leaves by degenerative leaves as a typical morphology during acclimatization.

An arrow indicates stacking of emerging leaves due to degeneration of outer leaves. Arrowheads indicate the release of stacking by removing degenerating parts of leaves.
