# Supplementary material for: Reconstructing tissue culture to improve Agrobacterium‐mediated transformation of maize
Source: Plant J. 2026 Oct 23;128(2):e71183. doi: 10.1111/tpj.71183 (PMC13691924; doi:10.1111/tpj.71183)
Supplement: Supplementary file 10 [file TPJ-128-0-s011.pdf]

with Carbenicillin (100 mg/L) **with Meropenem (25 mg/L)**

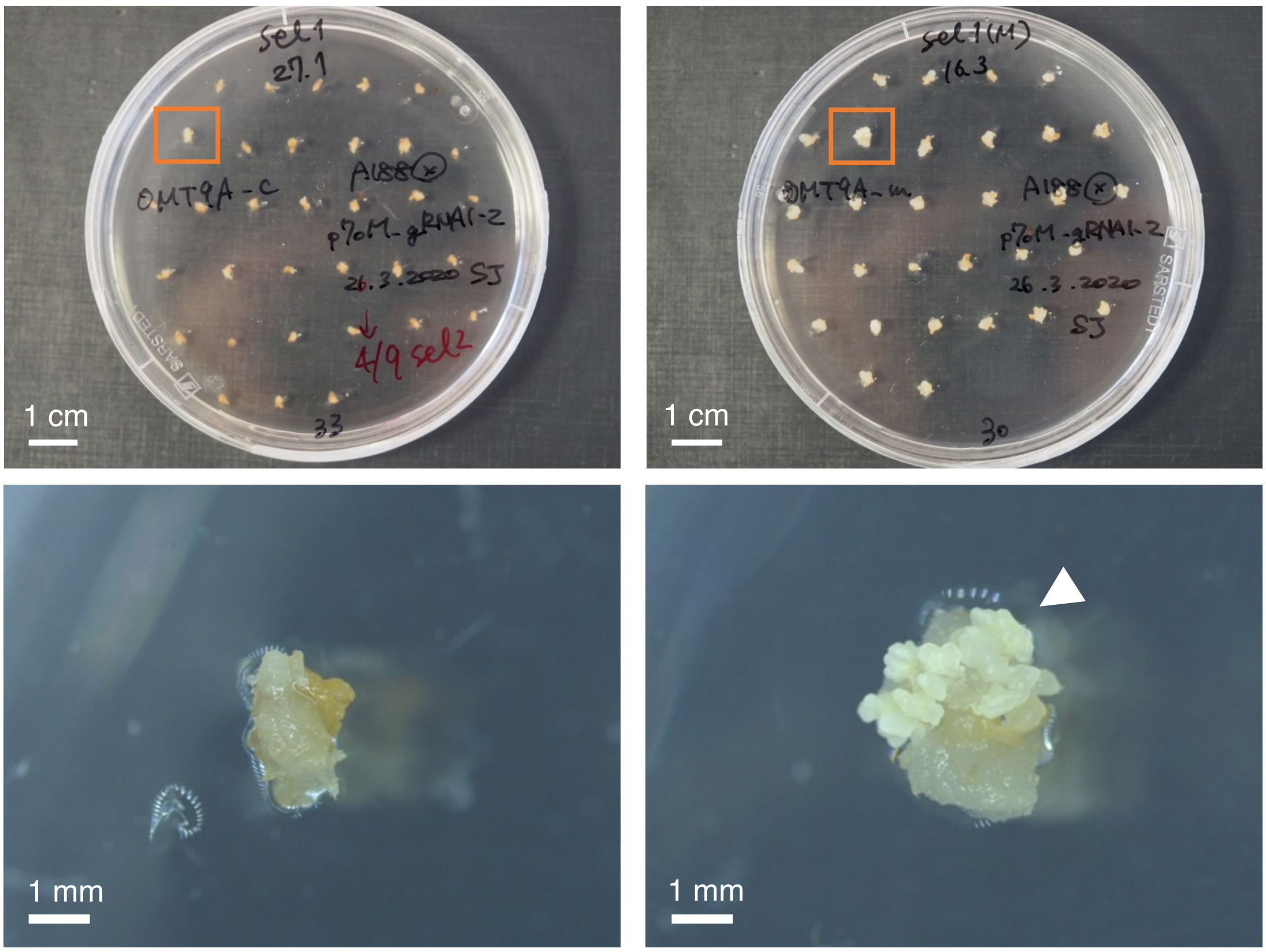

Supporting Figure S1
