# Supplementary material for: Reconstructing tissue culture to improve Agrobacterium‐mediated transformation of maize
Source: Plant J. 2026 Oct 23;128(2):e71183. doi: 10.1111/tpj.71183 (PMC13691924; doi:10.1111/tpj.71183)
Supplement: Supplementary file 11 [file TPJ-128-0-s012.pdf]

Supporting Experimental Procedure S2 (Methods S2)

TAFLP (T-DNA Amplified Fragment Length Polymorphism) protocol

Table of contents:

1. **Selection of restriction enzyme and designing of adapters and PCR primer** **pp. 3**
2. **Preparation of genomic DNA samples** **pp. 4-6**
3. **Adapter preparation, genomic DNA digestion and adapter ligation.** **pp. 7-9**
4. **Sequential nested PCRs** **pp. 10-13**

1. **Selection of restriction enzyme and designing of adapters and PCR primers**

1.1 The method relies on the use of specific restriction enzymes, of which restriction site has 4 core fixed bases in the middle and 2 outer purine/pyrimidine bases. Theoretically, this type of enzymes has one restriction site every 1 kbp (1024 bp) in average, suitable to detected amplicons by PCR and agarose gel electrophoresis.

1.2 An assay using **ApoI** is shown in this procedure, of which restriction sequence is 5'-R/AATTY-3'. Another possibility would be **NspI** (5'-RCATG/Y-3'), although the actual assay has not tested in our hands.

1.3 **IMPORTANT:** Check your T-DNA binary vector sequence, especially close to your Left-border and Right-border. If you find ApoI (or NspI) sites very close to LB or RB, like closer than 50 bp, you may not able to use this restriction enzyme because of the difficulties to design 3 nested primers in between.

1.4 In between restriction site from binary vector and LB/RB sequences, design 3 nested PCR primers (LB-nest1, LB-nest2 and LB-nest3, or those for RB), with Tm setting at 58-62 ºC. (when performing PCRs with annealing temperature at 57 ºC, see below for the details). Overlapping of nested primers with several base pair each other (less than 10-12 bp) would be acceptable, as long as those primer series are expected to work in nesting PCR manner. However, try to avoid to put primers very close to or overlapping to LB or RB sequence, with taking a consideration that some extent of sequence resection can take place when T-DNAs are inserted in the host genome.

1.5 The adapter sequences and primers for adapter side are as follows:

Adapters (with ApoI)	Sequence (5'- 3')
ApoI-AD-top	aatgatacggcgaccaccgagatctacacacactctttccctacacgacGCTCTTCCGATCTTGACGCC
ApoI-AD-bottom	aattGGCGTCAAGATCGGAAGAGCacacgtctgaactccagtcac
Primers	
AD-nest1	AATGATACGGCGACCACCGAG
AD-nest2	CCGAGATCTACACACACTCTTTCCC
AD-nest3	TTTCCCTACACGACGCTCTTCC

1.6 Use following adapter sequences in the case you are using NspI. Same AD- primers from ApoI-based assay (AD-nest1, AD-nest2 and AD-nest3) can be used.

Adapters (with NspI)	Sequence (5'- 3')
NspI-AD-top	aatgatacggcgaccaccgagatctacacacactctttccctacacgacGCTCTTCCGATCTTGACGCCcatg
NspI-AD-bottom	GGCGTCAAGATCGGAAGAGCacacgtctgaactccagtcac

2. **Preparation of genomic DNA samples.**

- DNA samples need to be not fragmented (preferentially to have clear bands at larger than 20-50 kbp by checking with agarose gel electrophoresis), RNaseA treated, and to have minimum concentration of 50 ng/µL, preferentially higher than 100 ng/µL (measured by Nanodrop).
- Extraction with column or magnetic bead kits would be perfect, however if you work with a lot of samples, follow the optimized method working with 96-well plate system below.

Equipment

- Centrifugation system compatible for 96-well plates (e.g.: Beckman coulter: Allegra X-15R centrifuge, equipping a swinging-bucket rotor SX4750)
- Mill mixer compatible for 96-well plates (e.g.: RETCH: MM300)
- Vortex mixer
- Water bath
- Dry incubation chamber set at 37 ºC.
- 96-well deep-well plate for lysate extraction (e.g., Greiner: Cat.No. 780201)
- Sealing mat for 96-well deep-well plate (e.g., Thermo: Cat. No. AB-0674)
- 2 mm ø stainless beads (larger diameter is also applicable)
- Full-skirted 96-well PCR plate for DNA sample preparation and storage (e.g., SARSTEDT: Cat.No., 72.1980, x2 per 96 samples)
- Compatible PCR sealing foil for 96-well PCR plate (e.g., Biozym: Cat. No. AS4TI-0500, x2 per 96 samples)
- (Preferable) Multichannel pipet for 10-100 µL (e.g.: Eppendorf: Cat.No., 3125000036)
- (Preferable) Solution Basin for multichannel pipette (e.g.: neoLab: Cat.No., 4-0015)

Reagents and enzymes

- Extraction buffer: 100mM Tris-HCl (pH7.5), 50mM EDTA (pH8.0), 500mM NaCl, 50mM Sodium Sulfate, and 0.5% SDS
- 5M Potassium Acetate
- Isopropanol (2-propanol)
- Absolute Ethanol (≥ 99.5 %)
- 70 % Ethanol
- 3M Sodium Acetate (pH=5.2)
- 1/5 TE buffer: 2mM Tris-HCl (pH=7.5) and 20 µM EDTA (pH=8.0)
- Elution buffer: 10mM Tris-HCl (pH=7.5)
- RNaseA (e.g., NewEnglandBiolabs: Cat.No., T3018L)

2.1. Collect young leaf material (~10 cm^2) into the wells filled with a stainless bead and 200 µL of Extraction Buffer. Seal the 96-well deep-well plate with a sealing mat tightly.

2.2. Grind young leaf material with a mill mixer, 2 times of 30/s frequency for 1 min.

2.3. Spin-down (x 5000 g, 2 min, at room-temperature) and deep-freeze plates (for 1 hour to overnight) with grind sample. This step is helpful to prevent contamination among samples caused by foaming of Extraction Buffer.

2.4. Incubate plates in a water bath at 65ºC for 1 hour.

2.5. Add 50 µL of 5M Potassium Acetate and vortex well, incubate at room temperature for 30 min (vortex every 10 min).

2.6. Centrifuge the plate, x5000 g, for 1 hr, at room temperature. Prepare 75 µL of Isopropanol in full-skirted 95-well PCR plates.

2.7. Carefully transfer 75 µL of supernatant into prepared Isopropanol. Few amounts of debris is acceptable. DO NOT mix with pipetting. After tight sealing with PCR sealing foil, perform gentle inverting of the plates to homogenize the mixture.

2.8. Incubate mixture for 5 min at room temperature, then centrifuge, x5000 g, 30 min, at room temperature. Discard supernatant by performing brief upside-down spin (immediately stop the centrifuge when it reaches x 8 g).

2.9. Add 150µL of 70% Ethanol to wash the pellet. Centrifuge and discard supernatants as the same way as in step 2.7.

2.10. Dry pellets in the 37ºC chamber (covered with plastic foil) for 1 hr.

2.11. Elute pellets in 30 µL of 1/5 TE buffer supplemented with 1 µg of RNase A. Perform vortex and spin down for several time and incubate at room temperature for 15 min. You can also pause here by leaving elutes in the -20 ºC freezer.

2.12. Perform centrifugation (x5000 g, 10 min, at room temperature) to precipitate debris. Prepare

47 µL of absolute ethanol with 150 mM Sodium Acetate (mix absolute ethanol and 3M Sodium Acetate in 19:1 ratio) in 96-well PCR plates and transfer 20 µL of supernatant. DO NOT mix with pipetting. After tight sealing, perform gentle inverting of plates to homogenize the mixture. Freeze mixture for 1 hr or for an overnight to perform ethanol precipitation of DNA.

2.13. Centrifuge x 5000 g, 30 min, at 4 ºC. Discard supernatant as the same way in step 2.8.

2.14. Add 150µL of 70 % Ethanol to wash the pellet. Centrifuge and discard supernatants as the same way in step 2.8. Dry pellet completely in 37ºC chamber (for 2 - 3 hrs). Elute pellets completely in 15 µL of Elution Buffer, by repeated vortex and spin down.

2.15 (Optional) If needed, check concentration and run on an agarose gel to check the quality of DNA samples. However, as long as the initial input amount of leaf material are nearly same, the yield can be nearly uniform. Perform dilution to 100 ng /µL with elution buffer. Now your DNA samples are ready to go the assay.

3. Adapter preparation, genomic DNA digestion and adapter ligation.

Equipment

- Centrifugation system compatible for 96-well plates (e.g.: Beckman coulter: Allegra X-15R centrifuge, equipping a swinging-bucket rotor SX4750)
- Thermal cycler compatible for 96-well plate system (e.g.: Thermo: SimpliAmp™ Thermal Cycler)
- Vortex mixer
- Dry incubation chamber set at 37 °C
- 96-well PCR plate to perform enzyme digestion and adapter ligation (e.g., StarLab: Cat.No., I1402-9800, x1 per 96 samples)
- PCR tubes to perform oligo hybridization (e.g., SARSTEDT: Cat.No., 72.991.002)
- Compatible PCR sealing foil for 96-well PCR plate (e.g., Biozym: Cat. No. AS4TI-0500)
- (Preferable) Multichannel pipet for 0.1-10 µL (e.g.: Eppendorf: Cat.No., 3125000010)

Reagents and enzymes

- 10x Hybridization buffer: 0.5M NaCl and 0.1M Tris-HCl (pH7.5)
- ApoI-HF (NewEnglandBiolabs: Cat.No., R3566L)
- 10x rCutSmart buffer (supplemented to ApoI-HF)
- 50 % (w/v) PEG3350 solution (made from Poly(ethylene glycol) average Mn 3350, powder, e.g., Sigma-Aldrich: Cat.No., 20244-500G)
- T4-DNA ligase (NewEnglandBiolabs: Cat.No., M0202L)
- 10x T4 DNA ligase buffer (supplemented to T4-DNA ligase)
- Water (PCR grade or ddH$_2$O)

3.1. Genome DNA digestion with ApoI.

Prepare following mixture in the new PCR plate, sealed with PCR foil tightly and incubate 37°C for at least 3 hrs.

Purified genome DNA (-100 ng/µL)	4 µL
10x rCutSmart buffer	1 µL
Water	4.88 µL
ApoI-HF	0.12 µL
Total	10 µL

3.2. During genome DNA digestion, prepare hybridized adapter for necessary amount as follows in the PCR tube.

<Adapter mixture for 96 samples>

ApoI-AD-top oligo (100 µM)	18.9 µL
ApoI-AD-bottom oligo (100 µM)	18.9 µL
10x Hybridization buffer	4.2 µL
Total	42 µL

Perform hybridization on thermal cycler (Program: Oligo-hyb)

95 ºC	5min
95 ºC to 8 ºC	| 1 min (-1 ºC/min) x87 cycles
8 ºC	hold

Keep this <Hybridized adapter> on ice until use.

3.3. Prepare Adapter-ligation premix as follows,

<Adapter ligation premix, for 1 sample, and for 96 sample (1 plate)>

10x T4 DNA ligase buffer (NEB)	1 µL	105 µL
PEG3850 (50% w/v)	1 µL	105 µL
Hybridized adapter (from Step 3.2)	0.4 µL	42 µL
Water (nuclease-free)	7.5 µL	787.5 µL
T4 DNA ligase	0.1 µL	10.5 µL
Total	10 µL	1050 µL

Pickup reaction plate from step 3.1 and add 10 µL of Adapter-ligation premix to each sample on ice. Mix well by vortex and spin down repeating at least 3 times.

IMPORTANT: DO NOT perform heat inactivation of ApoI enzyme, since ApoI needs to be still active following cyclic digestion-ligation step.

3.4. Perform cyclic digestion-ligation reaction on a thermal cycler. (Program: Goldengate-AD)

16 ºC	2 hrs	
16 ºC	30 min	|
37 ºC	10 min	| x 10cycle
65 ºC	20 min	

8 ºC hold

This reaction takes more than 9 hrs, would be an overnight reaction.

Keep the reaction as <adapter ligated sample>, go directly to 4. Sequential nested PCRs or freeze them at -20 oC until use.

4. Sequential nested PCRs

Equipment

- Centrifugation system compatible for 96-well plates (e.g.: Beckman coulter: Allegra X-15R centrifuge, equipping a swinging-bucket rotor SX4750)
- Thermal cycler compatible for 96-well plate system (e.g.: Thermo: SimpliAmp™ Thermal Cycler)
- Vortex mixer
- Agarose gel running system and documenting system
- 96-well PCR plate to perform PCR (e.g., StarLab: Cat.No., I1402-9800, x 3 per 96 samples)
- Compatible PCR sealing foil for 96-well PCR plate (e.g., Biozym: Cat. No. AS4TI-0500, x 3 per 96 samples)
- (Preferable) Multichannel pipet for 10-100 µL (e.g.: Eppendorf: Cat.No., 3125000036)
- (Preferable) Multichannel pipet for 0.5-10 µL (e.g.: Eppendorf: Cat.No., 3125000010)
- (Preferable) Solution Basin for multichannel pipette (e.g.: neoLab: Cat.No., 4-0015)

Reagents and enzymes

- DNA polymerase for versatile PCR genotyping system (e.g., Thermo: Cat.No., EP0704 and EP0714, see note below)
- 5x PCR enhancer: 750 mM trehalose, 0.1% nonacetylated BSA, 1% Tween-20 and 8.5 mM Tris-HCl (pH=8.0), from Samarakoon et al., (2013) doi: 10.3732/apps.1200236)
- dNTP mixture (2 mM each, made from Thermo: Cat.No., R0182)
- Water (PCR grade or ddH_2O)
- Agarose (e.g., Sigma: A9539-500G)
- 1x TAE buffer for agarose gel electrophoresis
- DNA ladder marker (e.g., Thermo: Cat.No., SM0331)

Note:

Regarding the PCR DNA polymerase throughout these nested PCR steps, higher grade of DNA polymerase can be used. However, you may need to optime cycle numbers, dilution factor and so on, depending on the efficiency of DNA polymerase you use. Here, a cheaper option with DreamTaq DNA polymerase (Thermo) is shown.

4.1. First round PCR (asymmetric)

Prepare following reaction and perform first PCR.

IMPORTANT: Use the primer concentration of <u>**AD-nest1 in 1 µM** (final conc. in the PCR reaction is 50 nM)</u>, is 10 times dilution compared to the other LB-nest1 primer. This is important to reduce the background noise of the final result.

When you are performing Right Border assay, replace primer LB-nest1 to RB-nest1.

PCR mixture (for 1 sample and for 96 sample (1 plate)):

Adapter-ligated sample	1 µL	
10x DreamTaq buffer	0.8 µL	84 µL
dNTP mixture (2 mM each)	0.8 µL	84 µL
5x PCR enhancer	1.6 µL	168 µL
Primer: AD-nest1 (**1 µM**)	0.4 µL	42 µL
Primer: LB-nest1 (10 µM)	0.4 µL	42 µL
Water	2.96 µL	311 µL
DreamTaq DNA polymerase	0.04 µL	4.2 µL
Total	8 µL	

PCR cycling:

95 ºC	2min	
95 ºC	30sec	|
57 ºC	30sec	| x 10 cycle
72 ºC	3min	|
16 ºC	hold	

After PCR, dilute the reaction to 10-fold, by adding 72 µL of water and mix well by vortex and spin down. Seal the plate tightly to prevent leaking and cross contamination.

4.2. Second round PCR (asymmetric)

Prepare following reaction and perform second PCR. Again, the primer concentration of **AD-nest2 in 1 µM** (final conc. in the PCR reaction is 50 nM).

When you are performing Right Border assay, replace primer LB-nest2 to RB-nest2.

PCR mixture (for 1 sample and for 96 sample (1 plate)):

1/10 diluted sample from step 4.1	0.8 µL	
10x DreamTaq buffer	0.8 µL	84 µL
dNTP mixture (2 mM each)	0.8 µL	84 µL
5x PCR enhancer	1.6 µL	168 µL
Primer: AD-nest2 (**1 µM**)	0.4 µL	42 µL
Primer: LB-nest2 (10 µM)	0.4 µL	42 µL
Water	3.16 µL	332 µL
DreamTaq DNA polymerase	0.04 µL	4.2 µL
Total	8 µL	

PCR cycling:

95 ºC	2min	
95 ºC	30sec	|
57 ºC	30sec	| x 10 cycle
72 ºC	3min	|
16 ºC	hold	

After PCR, dilute the reaction to 10-fold, by adding 72 µL of water and mix well by vortex and spin down. Seal the plate tightly to prevent leaking and cross contamination.

4.3. Third round PCR (symmetric)

Prepare following reaction and perform third PCR. This time, the primer concentration on the adapter side (AD-nest3) is same as the left border primer (**10 µM**). For the convenience, dye-premixed PCR buffer is preferable if it's available. The cycle number for this round is increased to 35 cycles.

When you are performing Right Border assay, replace primer LB-nest3 to RB-nest3

PCR mixture (for 1 sample and for 96 sample (1 plate)):

1/10 diluted sample from step 4.2	0.8 µL	
10x DreamTaq Green buffer	0.8 µL	84 µL
dNTP mixture (2 mM each)	0.8 µL	84 µL
5x PCR enhancer	1.6 µL	168 µL
Primer: AD-nest3 (**10 µM**)	0.4 µL	42 µL
Primer: LB-nest3 (10 µM)	0.4 µL	42 µL
Water	3.16 µL	332 µL
DreamTaq DNA polymerase	0.04 µL	4.2 µL
Total	8 µL	

PCR cycling:

95 ºC	2min	
95 ºC	30sec	|
57 ºC	30sec	| x 35 cycle
72 ºC	3min	|
16 ºC	hold	

Run PCR product on 1.0 % agarose gel (with 1x TAE buffer system) to see specific amplification of T-DNA border. Run DNA ladder makers on the both end of the gel. Run the products for 5-6 cm, or for 40 min with constant 130 V condition.
